# A genetic cell context-dependent role for ZEB1 in lung cancer

**DOI:** 10.1038/ncomms12231

**Published:** 2016-07-26

**Authors:** Ting Zhang, Lixia Guo, Chad J. Creighton, Qiang Lu, Don L. Gibbons, Eunhee S. Yi, Bo Deng, Julian R. Molina, Zhifu Sun, Ping Yang, Yanan Yang

**Affiliations:** 1 Thoracic Disease Research Unit, Division of Pulmonary and Critical Care Medicine, Cancer Center and College of Medicine, Mayo Clinic, 200 First Street SW, Stabile Building Room st8-08 Rochester, Minnesota 55905, USA; 2 Department of Biochemistry and Molecular Biology, Cancer Center and College of Medicine, Mayo Clinic, Rochester, Minnesota 55905, USA; 3 Dan Duncan Cancer Center, Baylor College of Medicine, Houston, Texas 77030, USA; 4 Division of Epidemiology, Department of Health Sciences Research, Mayo Clinic College of Medicine, Rochester, Minnesota 55905, USA; 5 Department of Thoracic Head and Neck Medical Oncology, M.D. Anderson Cancer Center, Houston, Texas 77030, USA; 6 Department of Molecular and Cellular Oncology, M.D. Anderson Cancer Center, Houston, Texas 77030, USA; 7 Department of Laboratory Medicine and Pathology, Mayo Clinic, Rochester, Minnesota 55905, USA; 8 Thoracic Surgery Department, Institute of Surgery Research, Daping Hospital, Third Military Medical University, Chongqing 400042, China; 9 Division of Medical Oncology, Mayo Clinic, Rochester, Minnesota 55905, USA; 10 Division of Biomedical Statistics and Informatics, Mayo Clinic, Rochester, Minnesota 55905, USA

## Abstract

The Zinc-finger E-box-binding Homeobox-1 (ZEB1) is a transcription factor that promotes epithelial–mesenchymal transition (EMT) and acts as an oncogene in *KRAS*-mutated lung cancer models. Here we report that ZEB1 exerts the opposite effect in *EGFR*-mutated lung cancer cells, where it suppresses growth by increasing microRNA-200 targets to antagonize ERBB3, a driver of mutant *EGFR*-dependent cell growth. Among these targets, NOTCH1 represses *ERBB3* promoter activity and the expression of ERBB3. Furthermore, we find that EGFR inhibitor treatment, which inhibits the growth of *EGFR*-mutated cells, induces ZEB1. Despite its growth-inhibiting effect, EGFR inhibitor-induced ZEB1 strongly promotes EMT-dependent resistance to EGFR inhibitors partially through NOTCH1, suggesting a multifunctional role for NOTCH1 in *EGFR*-mutated cells. These results support a previously unrecognized genetic cell context-dependent role for ZEB1 and suggest that NOTCH1 may be a useful target for treating resistance to EGFR inhibitors, especially EMT-driven resistance.

## Abstract (web summary)

ZEB1 is a driver of epithelial-to-mesenchymal transition that usually promotes lung cancer in the context of KRAS mutation. Here, the authors uncover a growth suppressive role for ZEB1 in *EGFR* mutant lung adenocarcinoma, thus elucidating the context dependent function of this protein.

## Graphical abstract

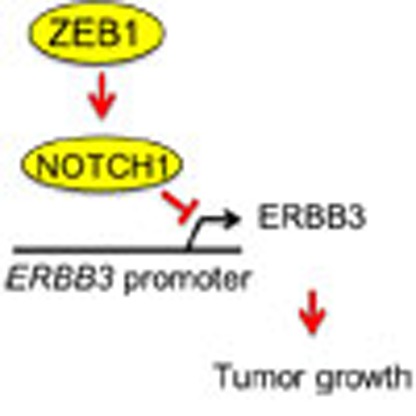

Cancer development is associated with complex genetic changes, including mutations of oncogenes and tumour suppressors. Some oncogenic mutations occur in a mutually exclusive manner in specific types of cancers, such as the *KRAS* and *EGFR* mutations in lung cancer^1^,^2^. The biologic basis for this phenomenon is not well understood. It is possible that cellular processes regulated by mutant *KRAS* or *EGFR* may have contradictory functions that exclude each other. Clinically, *KRAS* mutations in lung cancer are associated with resistance to epidermal growth factor receptor (EGFR) inhibitors^1^,^2^.

ZEB1 is a zinc-finger E-box-binding homeobox protein that induces epithelial–mesenchymal transition (EMT), a reversible process with multiple roles in cancer development^3^,^4^. Recent studies have shown that ZEB1 acts as an oncogene in invasive and metastatic lung cancer cells, in which ZEB1-induced EMT promotes the loss of epithelial cell polarity and adhesion, induces cytoskeleton remodelling and drives growth, migration, invasion and metastasis^5^,^6^,^7^,^8^,^9^,^10^,^11^. The role of ZEB1 in early-stage lung cancer remains poorly explored. A recent report showed that ZEB1 is required for mutant *KRAS*-driven lung tumour initiation and progression^12^. Notably, most of these studies address the role of ZEB1 by using cells or mouse models that express mutant *KRAS*, without exploring whether ZEB1 has different roles in lung cancer cells without *KRAS* mutations.

Herein, we report an unexpected finding that ZEB1 plays an opposite role in *EGFR*-mutated lung cancer cells by acting as a suppressor of cell growth. We show that ZEB1 transcriptionally represses microRNA-200 (miR-200) to increase several miR-200 target genes that inhibit the receptor tyrosine kinase ERBB3, which is essential for growth of *EGFR*-mutated lung cancer cells^13^,^14^,^15^,^16^,^17^. *In vivo*, ERBB3 is repressed in lung adenocarcinoma tissue with elevated ZEB1 expression. Furthermore, we find that the growth inhibition induced by an EGFR inhibitor, gefitinib, is associated with strong induction of ZEB1, which in turn promotes EMT-dependent resistance to gefitinib. Mechanistically, we show that NOTCH1 is the mediator of ZEB1 that transcriptionally inactivates *ERBB3* and promotes EMT and resistance. Thus, the biologic functions of ZEB1 and NOTCH1 are context dependent. Within the context that EGFR is inhibited, ZEB1 and NOTCH1 exert an additional role that may contribute to the survival of a subset of *EGFR*-mutated cells by promoting EMT. As there are no clinically effective drugs for EGFR inhibitor-resistant lung tumours with EMT features, these findings warrant future studies that test the efficacy of combined use of NOTCH1 and EGFR inhibitors in the treatment of such resistant lung tumours.

## Results

### ZEB1 was repressed in lung adenocarcinomas

Several E-box-binding transcription repressors, including ZEB, SNAIL and TWIST, are known to promote EMT by repressing epithelial genes (for example, *CDH1*) and by increasing mesenchymal genes (for example, *VIM*)^18^,^19^,^20^. To explore the role of such EMT-promoting factors in lung carcinogenesis, we mined the Oncomine data sets of lung adenocarcinomas (nine data sets from www.oncomine.org) for alterations in *CDH1*, *VIM*, *ZEB1*, *ZEB2*, *SNAI1*, *SLUG* (also known as *SNAI2*) and *TWIST* (Table 1). We found that lung adenocarcinomas exhibited an epithelial *CDH1*^high^/*VIM*^low^ gene expression profile compared with normal lung tissues, demonstrating higher *CDH1* in eight of nine data sets but lower *VIM* in all nine data sets. In five of six data sets containing ZEB1 expression data, tumours had lower ZEB1, which was associated with the epithelial-like phenotype of the tumours and supported the role of ZEB1 as a driver of EMT. In contrast, expression levels of the remaining factors were not different between tumours and normal tissues (*ZEB2*, *SNAI1* and *SLUG*), or even higher in tumours (*TWIST*), implying that these factors may not be the primary regulators of EMT in lung adenocarcinomas.

**Figure 1:**
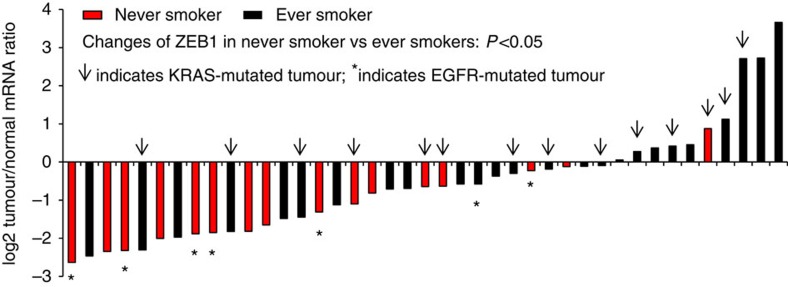
The changes of *ZEB1* expression in lung adenocarcinomas compared with normal lung tissues are associated with smoking status. The mRNA levels of *ZEB1* in 41 pairs of lung adenocarcinomas and normal lung tissues (Supplementary Table 1) were quantified by qPCR and normalized to that of *L32* (internal control). The changes in *ZEB1* expression were expressed as logged (log2) ratios of ZEB1 level in tumours versus normal tissues. Note: arrow indicates KRAS-mutated tumours and * indicates EGFR-mutated tumours.

Although the above observations raised the possibility that ZEB1 may be more related to EMT in lung adenocarcinoma compared with other E-box-binding factors, they also suggested a growth-suppressor role for ZEB1, which contradicts previous reports supporting an oncogenic role of ZEB1 in *KRAS*-mutated lung cancer^5^,^6^,^7^,^8^,^9^,^10^,^11^,^12^. To validate these observations, we quantified the expression of *ZEB1*, *CDH1* and *VIM* for 41 paired samples of lung adenocarcinoma and adjacent normal lung tissues (Supplementary Table 1). We found that the changes in gene expression in tumours versus normal tissues were similar to what we observed in the Oncomine data sets, namely that tumours had an epithelial-like *CDH1*^high^/*VIM*^low^ phenotype, which was associated with lower ZEB1 expression (Supplementary Table 2). Consistently, immunohistochemical staining results showed that lung adenocarcinoma cells expressed high levels of E-cadherin but low to non-detectable levels of Vimentin and ZEB1 (Supplementary Fig. 1a). Notably, ZEB1 was repressed in nearly all tumours from never smokers (15 out of 16) (Fig. 1), a significantly higher frequency of repression than that observed in smokers (Fisher's exact text, *P*<0.05) (Fig. 1). Given that the incidence of certain somatic driver mutations in lung adenocarcinomas differs in smokers and non-smokers, for instance, lung tumours of never smokers have more *EGFR* mutations but fewer *KRAS* mutations compared with tumours from smokers^21^,^22^, we hypothesize that ZEB1 exerts a growth suppressive role that is genetic context dependent.

Sequencing of the 41 lung adenocarcinomas showed that 14 tumours had *KRAS* mutations, and that 7 tumours had *EGFR* mutations (Fig. 1 and Supplementary Table 3). Of the ten tumours with higher ZEB1, five (50%) had *KRAS* mutations (Fig. 1). This percentage was higher than that of tumours with lower ZEB1 (9 *KRAS*-mutated tumours in a total of 31 tumours; 29%) (Fig. 1), but the association between *KRAS* mutations and ZEB1 expression changes was not statistically significant (Fisher's exact test, *P*=0.11). In contrast, all seven *EGFR*-mutated tumours had lower ZEB1 (Fig. 1) and the levels of ZEB1 were significantly lower in *EGFR* mutant tumours than that in *EGFR* wild-type tumours (Student's *t*-test *P*=0.027) or in *KRAS* mutant tumours (Student's *t*-test *P*=0.013). In support of these findings, most *EGFR*-mutated lung adenocarcinomas from The Cancer Genome Atlas (TCGA) data set (7 of 9 paired lung adenocarcinoma and normal lung tissues) or from previous reports (12 of 13 paired lung adenocarcinoma and normal lung tissues; reported in refs 23, 24) had lower ZEB1 compared with paired normal lung tissues (Supplementary Fig. 1b,c). In most Oncomine data sets with lower tumour ZEB1 (Table 1), the expression levels of both EGFR and KRAS were marginally higher in tumours compared with paired normal lung tissues (Supplementary Table 4). Collectively, these results suggest that *EGFR* mutations correlate with the loss of ZEB1 in lung adenocarcinomas.

### ZEB1 oppositely regulated KRAS and EGFR mutant cell growth

**Figure 2:**
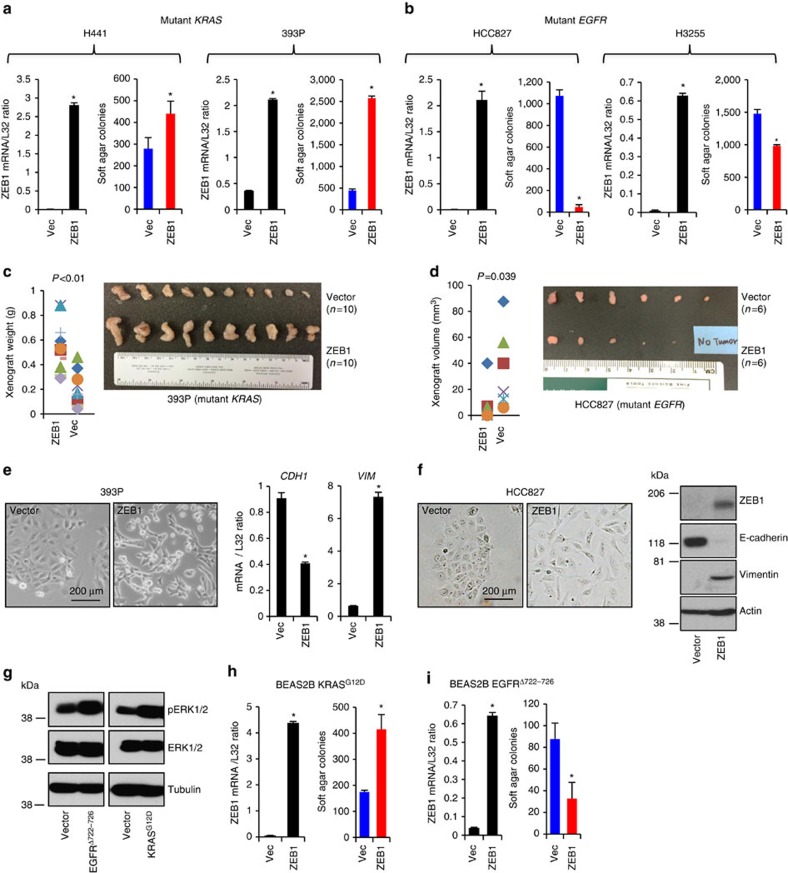
ZEB1 distinctly regulates soft agar and xenograft tumour growth of lung cancer cells expressing mutant *KRAS* or mutant *EGFR*. ZEB1 distinctly regulates soft agar growth of lung cancer cells expressing mutant *KRAS* (**a**, H441 and 393P cells) or mutant *EGFR* (**b**, HCC827 and H3255 cells). ZEB1 expression was quantified by qPCR (left bar charts in **a** and **b**) and numbers of soft agar colonies formed by 1 × 10^4^ cells in six-well plates (triplicates; mean plus s.d.) in 3 weeks were counted (coloured bar charts in **a** and **b**). * indicates *t*-test *P*<0.05. ZEB1 promotes KRAS-mutated 393P xenograft tumour growth (**c**) but inhibits EGFR-mutated HCC827 xenograft tumour growth (**d**). Xenograft tumours were weighed and measured (scatter plots in **c** and **d**) and photographed (pictures in **c** and **d**). ZEB1 promotes EMT of both 393P and HCC827 cells. EMT was examined by morphological changes (pictures on left; scale bar: 200 μm) and by qPCR for *CDH1* and *VIM* (**e**, bar charts; triplicates; mean plus s.d.) or western blotting for E-cadherin and Vimentin (**f**, gels). (**g**) Western blotting for BEAS2B cells expressing *KRAS*^*G12D*^ or *EGFR*^*Δ722–726*^, or a control empty pcDNA3.1 vector. ZEB1 distinctly regulates soft agar growth of BEAS2B cells expressing *KRAS*^*G12D*^ (**h**) or *EGFR*^*Δ722-726*^ (**i**). ZEB1 expression was quantified by qPCR (left bar charts in **h** and **i**) and numbers of soft agar colonies formed by 1 × 10^4^ cells in six-well plates (triplicates; mean plus s.d.) in 3 weeks were counted (coloured bar charts in **h** and **i**). * indicates *t*-test *P*<0.05.

Next, we expressed ZEB1 in lung adenocarcinoma cells, including H441, 393P, HCC827 and H3255 cells. H441 and 393P are *KRAS* mutated, and HCC827 and H3255 are *EGFR* mutated. None of them expressed endogenous ZEB1 (Supplementary Fig. 2a). We found that ZEB1 promoted *KRAS*-mutated cell growth (Fig. 2a) but inhibited *EGFR*-mutated cell growth (Fig. 2b). Consistently, ZEB1 also promoted *KRAS*-mutated xenograft tumour growth (Fig. 2c) but suppressed *EGFR*-mutated xenograft tumour growth (Fig. 2d). However, ZEB1 induced EMT in both of them (Fig. 2e,f). These results suggest that ZEB1 plays distinct roles in cells with *EGFR* or *KRAS* mutations, and that such roles are likely to be independent of EMT.

We realized that additional gene mutations may coexist with *KRAS* or *EGFR* mutations in cancer cells and may also affect the function of ZEB1. To address this point, we expressed *KRAS*^*G12D*^ or *EGFR*^*Δ722–726*^ in BEAS2B cells, a lung epithelial cell line. Western blotting of transfectants showed that *KRAS*^*G12D*^ slightly increased (by 27%) and *EGFR*^*Δ722–726*^ slightly decreased (by 16%) ZEB1 expression (Supplementary Fig. 2b), and both mutants activated the phosphorylation of extracellular regulated, mitogen-activated proteinkinases, indicating that the transfected genes were functional (Fig. 2g). Similar to the findings from cancer cells, ZEB1 also oppositely regulated the growth of BEAS2B cells expressing *KRAS*^*G12D*^ or *EGFR*^*Δ722-726*^ (Fig. 2h,i).

Collectively, our findings are consistent with the previously reported oncogenic role of ZEB1 in *KRAS*-mutated cells^5^,^6^,^7^,^8^,^9^,^10^,^11^,^12^. Beyond this, they provide evidence of a completely new function of ZEB1 as a growth suppressor in *EGFR*-mutated cells.

### ZEB1 repressed ERBB3 through miR-200c repression

**Figure 3:**
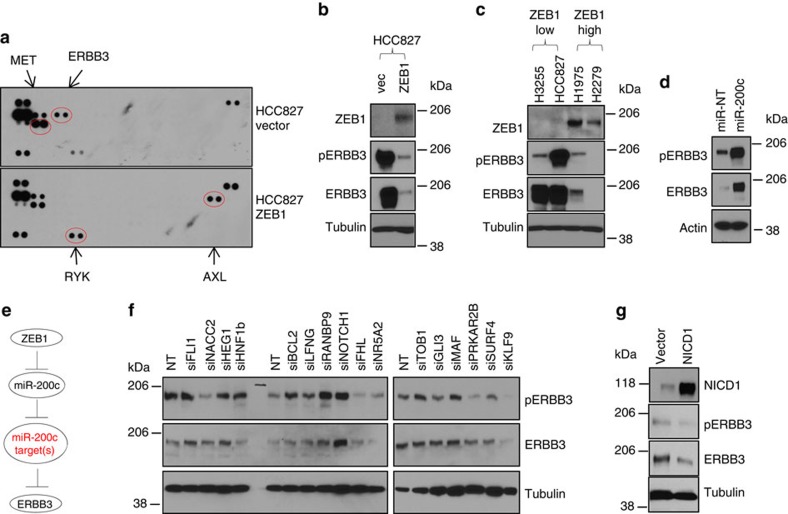
ZEB1 suppresses *ERBB3* through miR-200c targets. (**a**) Phospho-receptor tyrosine kinase (RTK) array for HCC827 expressing ZEB1 or an empty pcDNA3.1 vector. (**b**) Western blotting of ZEB1, phospho-ERBB3 (pERBB3), ERBB3 and Tubulin for HCC827 cells expressing ZEB1 or the pcDNA3.1 vector. (**c**) Western blotting of ZEB1, pERBB3, ERBB3 and Tubulin for a panel of four *EGFR*-mutated human lung cancer cell lines. (**d**) miR-200c increased both pERBB3 and ERBB3. (**e**) Schematic for hypothetical role of miR-200c targets in ERBB3 inhibition. (**f**) The effect of individual knockdown of miR-200c targets on the expression and phosphorylation of ERBB3 in HCC827 cells expressing ZEB1. (**g**) Western blotting of NICD1, pERBB3, ERBB3 and Tubulin for H4006 cells transiently transfected with NICD1 or an empty vector (p3XFLAGCMV).

To understand the molecular basis underlying this new function of ZEB1, we performed a phospho-receptor tyrosine kinase array for *EGFR*-mutated HCC827 cells expressing ZEB1. We found that ZEB1 suppressed phosphorylation of ERBB3 and MET but promoted phosphorylation of RYK and AXL (Fig. 3a). Next, we focused on ERBB3, because a number of studies have described an essential role for ERBB3 in mutant EGFR-dependent lung cancer cell growth^13^,^14^,^15^,^16^,^17^. For instance, ERBB3 inactivation not only inhibits *EGFR*-mutated cell growth *in vitro* (including HCC827 cells), but also suppresses mutant *EGFR*-driven lung tumour growth in a mouse model^13^,^14^. Western blotting showed that ZEB1 suppressed the expression of ERBB3 (Fig. 3b). In a panel of EGFR-mutated cells, ERBB3 was higher in cells expressing low ZEB1 (Fig. 3c). Thus, we concluded that ZEB1 suppresses *EGFR*-mutated cell growth by repressing ERBB3. The clinical relevance of these findings is further supported by an analysis of The Cancer Genome Atlas (TCGA) (www.cancergenome.nih.gov) lung adenocarcinoma data set (*n*=510), in which ZEB1 negatively correlated with ERBB3 (Pearson's *R*=−0.24, *P*=6.94E−08). We noted that the level of phosphorylated ERBB3 was low in one ZEB1^low^ cell line, H3255, in which non-phosphorylated ERBB3 was highly expressed (Fig. 3c), suggesting that the expression and phosphorylation of ERBB3 can be regulated through differential mechanisms.

Next, we explored the mechanism whereby ZEB1 repressed ERBB3. As ZEB1 acts as a transcription repressor^18^,^19^, we first tested whether ZEB1 directly represses ERBB3 transcription. Our results (Supplementary Fig. 3a) showed that ZEB1 did not inhibit the activity of an ERBB3 promoter reporter (it actually slightly increased the ERBB3 promoter reporter activity), indicating that ZEB1 does not directly repress ERBB3 transcription. Recent findings have shown that transcriptional inactivation of miR-200 by ZEB1 is critical for its biologic functions, for example, EMT and invasion^25^,^26^. As such, we posited that ZEB1 repressed ERBB3 by inactivating miR-200. In support of this idea, ZEB1 strongly decreased miR-200c (Supplementary Fig. 3b) and re-expression of miR-200c dramatically restored ERBB3 expression and phosphorylation (Fig. 3d), and promoted cell growth (Supplementary Fig. 3c). Given that microRNAs are negative regulators of gene expression, we postulated that miR-200c represses its target genes to indirectly promote ERBB3 expression and phosphorylation (Fig. 3e). To identify potential targets, we performed RNA sequencing of HCC827 cells expressing ZEB1 (Supplementary Data 1) and found that 87 predicted miR-200c targets were increased by ZEB1 (fold change >1.5 and *P*<0.05) (Supplementary Fig. 4a). The 33′-untranslational region (3′-UTR) of each of these genes has at least one specific binding sequence for miR-200c (www.targetscan.org).

### MiR-200c targets suppressed ERBB3

**Figure 4:**
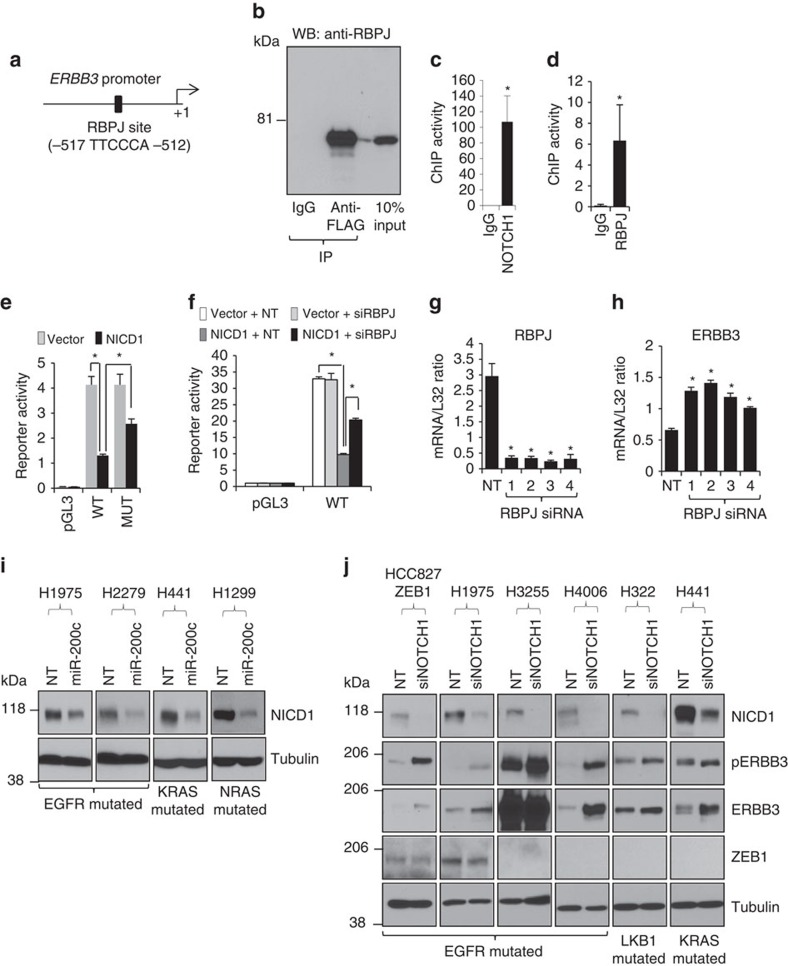
NICD1 and RBPJ regulate ERBB3 promoter. (**a**) Schematic for the *ERBB3* promoter containing a putative RBPJ-binding site. (**b**) Immunoprecipitation result showing NICD1 bound to RBPJ in H1299 lung cancer cells. H1299 cells were transiently transfected with FLAG-tagged NICD1, which were immunoprecipitated by anti-FLAG. Both IgG immunoprecipitates and 10% non-immunoprecipitated cell lysate were included in the RBPJ western blotting as controls. ChIP assays result showing that both NOTCH1 (**c**) and RBPJ (**d**) directly bound to the RBPJ site on *ERBB3* promoter in H1299 cells (triplicates; mean plus s.d.). * indicates *t*-test *P*<0.05. Mutation of the RBPJ binding site (**e**) or knockdown of RBPJ (**f**) suppressed the inhibitory effect of NICD1 on the *ERBB3* promoter activity (triplicates; mean plus s.d.). * indicates *t*-test *P*<0.05. qPCR of RBPJ (**g**) and ERBB3 (**h**) for H322 lung adenocarcinoma cells transfected with control NT non-targeting siRNA or RBPJ siRNAs (triplicates; mean plus s.d.). * indicates *t*-test *P*<0.05. (**i**) Western blotting of NICD1 and Tubulin for lung cancer cells transiently transfected with miR-200c or a control non-targeting microRNA (NT). (**j**) Western blotting of ZEB1, NICD1, pERBB3, ERBB3 and Tubulin for lung cancer cells transiently transfected with NOTCH1 siRNA or a control non-targeting siRNA (NT).

We randomly selected 16 of the 87 predicted miR-200c targets, individually transfected their small interfering RNAs (siRNAs) (Supplementary Fig. 4b), and performed western blotting. The results showed that knockdown of NOTCH1, but not any of the other 15 genes, strongly promoted both expression and phosphorylation of ERBB3 (Fig. 3f). On the contrary, expression of NICD1 abrogated ERBB3 expression (Fig. 3g and Supplementary Fig. 4c). Given that NICD1 is a transcription factor^27^, we posited that it transcriptionally represses *ERBB3*. In support of this idea, knockdown of NOTCH1 increased not only the protein but also the messenger RNA levels of ERBB3 (Supplementary Fig. 4d,e). Furthermore, γ-secretase inhibitors (DAPT and BMS-708163) induced ERBB3, mimicking the effect of NOTCH1 knockdown and the expression of NICD1 completely abolished the effect of γ-secretase inhibitors (Supplementary Fig. 4c), suggesting that NICD1 is both necessary and sufficient for ERBB3 repression. Notably, NICD1 complexes with transcription co-factors to form a transcription-regulating complex that directly binds to target gene promoters. Indeed, analysis of the *ERBB3* promoter (Fig. 4a) revealed a previously untested binding site for RBPJ, a well-characterized co-factor of NICD1 that plays an essential role in NOTCH1-dependent transcriptional regulation^27^. Our immunoprecipitation–western blotting experiment confirmed that NICD1 bound to RBPJ in lung cancer cells (Fig. 4b). By performing chromatin immunoprecipitation (ChIP) assays, we found that both NICD1 and RBPJ bound to this RBPJ site (Fig. 4c,d). Next, we performed luciferase promoter reporter assays and found that NICD1 inhibited the *ERBB3* promoter and either mutation of the RBPJ site or knockdown of RBPJ significantly suppressed the effect of NICD1 (Fig. 4e,f). Similar to the effect of NOTCH1 depletion, knockdown of RBPJ also promoted ERBB3 expression (Fig. 4g,h).

To further validate these results, we knocked down ZEB1 and found that miR-200c was increased and NOTCH1 was decreased (Supplementary Fig. 4f,g), supporting our finding that ZEB1 promoted NOTCH1 through repression of miR-200c. We also expressed miR-200c or NOTCH1 siRNA in multiple lung cancer cells with different oncogenic backgrounds. In nearly all these cells, miR-200c expression decreased NICD1 (Fig. 4i) and knockdown of NOTCH1 promoted both expression and phosphorylation of ERBB3 (Fig. 4j). Notably, the effect of miR-200c or NOTCH1 siRNA was not limited to *EGFR*-mutated cells, suggesting that miR-200c regulates a universal and conserved NOTCH1-ERBB3 signalling axis in lung cancer cells.

**Figure 5:**
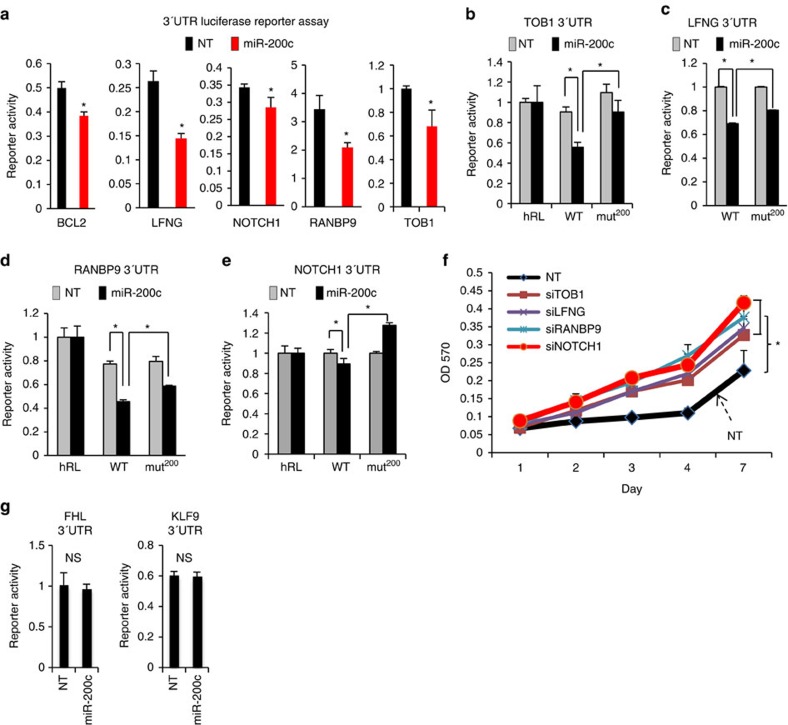
The validation of ERBB3-regulating genes as miR-200c targets. (**a**) 3′-UTR reporter assays for H1299 cells transiently co-transfected with miR-200c and the indicated genes' 3′-UTR reporter plasmids. (triplicates; mean plus s.d.). * indicates *t*-test *P*<0.05. Mutation of the miR-200c-binding site abrogates the inhibitory of miR-200c on the activity of 3′-UTRs of TOB1 (**b**), LFNG (**c**), RANBP9 (**d**) and NOTCH1 (**e**). (triplicates; mean plus s.d.). *indicates *t*-test *P*<0.05. (**f**) MTT assay for HCC827-ZEB1 cells transiently transfected with siRNAs against NOTCH1, TOB1, LFNG, RANBP9 or a control non-targeting siRNA (N.T.). (triplicates; mean plus s.d.). * indicates *t*-test *P*<0.05. (**g**) 3′-UTR reporter assays for H1299 cells transiently co-transfected with miR-200c and the indicated genes' 3′-UTR reporter plasmids. N.S., not statistically significant (*t*-test *P*>0.05).

We further observed that knockdown of several other genes, including *BCL2*, *RANBP9*, *LFNG*, *TOB1* and *MAF*, did not significantly affect ERBB3 expression but did promote its phosphorylation to varying extents (Fig. 3f). To determine whether these ERBB3 phosphorylation-regulating genes, as well as NOTCH1, are direct targets of miR-200c, we cloned the 3′-UTRs of *BCL2*, *RANBP9*, *LFNG*, *TOB1* and *NOTCH1* into a luciferase-conjugated 3′-UTR reporter plasmid^5^. We found that miR-200c suppressed all these 3′-UTRs (Fig. 5a) and mutation of their miR-200c binding sequences suppressed the effect of miR-200c (Fig. 5b–e). Consistently, knockdown of these genes promoted cell growth (Fig. 5f), mimicking the effect of miR-200c expression (Supplementary Fig. 3c). We also noted that the effect of miR-200c on NOTCH1 3′-UTR luciferase reporter activity was modest. However, miR-200c significantly repressed endogenous NOTCH1 expression in multiple lung cancer cells (Fig. 4i), suggesting that the regulation of NOTCH1 3′-UTR by miR-200c was critical for endogenous NOTCH1 expression. To examine the role of the ERBB3-regulating miR-200c target genes in tumour growth, we generated *EGFR*-mutated H1975 cell transfectants that stably express short hairpin RNAs against TOB1 (Supplementary Fig. 5a). We found that the knockdown of TOB1 alone moderately (*P*=0.074) increased xenograft tumour growth (Supplementary Fig. 5b,c), suggesting that these ERBB3-regulating genes may collaboratively regulate tumour growth *in vivo*.

In contrast to the effect of above-mentioned genes, knockdown of several other genes, including *FHL*, *KLF9*, *NACC2* and *PRKAR2B*, decreased ERBB3 phosphorylation (Fig. 3f). We also cloned the 3′-UTRs of FHL and KLF9, but the reporter assay results showed that they were not regulated by miR-200c (Fig. 5g). Collectively, our results suggest that miR-200c acts as an activator of ERBB3 and multiple mechanisms are likely to be involved in the regulation of ERBB3 by miR-200c.

### NOTCH1 drove EMT-dependent gefitinib resistance

**Figure 6:**
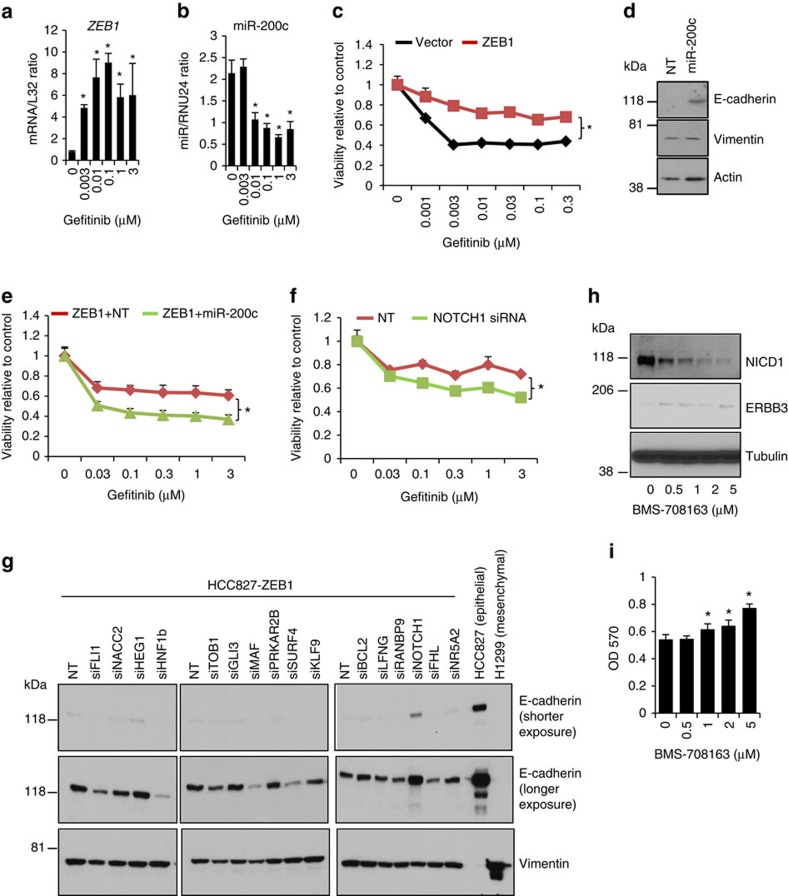
NOTCH1 regulates EMT and gefitinib resistance. HCC827 cells were treated with or without gefitinib for 2 weeks and the expression of *ZEB1* mRNA (**a**) and miR-200c (**b**) was quantified by qPCR and normalized to that of internal controls (*L32* for mRNA and RNU24 for microRNA) (triplicates; mean plus s.d.). * indicates *t*-test *P*<0.05. (**c**) MTT assay for HCC827 transfectants treated with or without gefitinib for 3 days (triplicates; mean plus s.d.). * indicates one-way analysis of variance (ANOVA) test, *P*<0.05. (**d**) Western blotting of E-cadherin, Vimentin and Actin for HCC827-ZEB1 cells transiently transfected with miR-200c or a control non-targeting microRNA (N.T.). (**e**) MTT assay for HCC827ZEB1 cells transiently transfected with miR-200c or a control non-targeting microRNA (N.T.) (triplicates; mean plus s.d.). * indicates one-way ANOVA test, *P*<0.05. (**f**) MTT assay for HCC827ZEB1 cells transiently transfected with NOTCH1 siRNA or a control non-targeting siRNA (NT) (triplicates; mean plus s.d.). * indicates one-way ANOVA test, *P*<0.05. (**g**) Western blotting of E-cadherin and Vimentin for HCC827-ZEB1 cells transiently transfected with siRNAs against miR-200c targets. Epithelial-like parental HCC827 cells and mesenchymal-like H1299 cells are included as positive controls that highly express E-cadherin and Vimentin, respectively. (**h**) Western blotting for HCC827GR cells treated with or without BMS-708163. (**i**) MTT assay for HCC827GR cells treated with or without BMS-708163 (triplicates; mean plus s.d.). * indicates *t*-test *P*<0.05.

Both EMT and ERBB3 are implicated in the acquisition of resistance to EGFR inhibitors, for example, gefitinib^13^,^28^. As ZEB1 strongly regulates both of them (Figs 2 and 3), we suspected that ZEB1 and its mediators (miR-200c and its targets) are involved in this process. Using gefitinib-sensitive HCC827 cells as a model, we found that chronic treatment of gefitinib strongly induced ZEB1 expression (Fig. 6a), accompanied by the loss of miR-200c (Fig. 6b) and an EMT phenotype (Supplementary Fig. 6). Despite its growth inhibitory effect (Fig. 2), ZEB1 dramatically drove resistance to gefitinib (Fig. 6c). As the expression of miR-200c partially reversed both EMT (Fig. 6d) and resistance (Fig. 6e), we screened the 16 miR-200c targets to identify resistance-related genes that may have therapeutic potential. The result revealed that knockdown of NOTCH1 partially reversed gefitinib resistance to an extent comparable to that of miR-200c (Fig. 6f). Knockdown of several other genes, including *NR5A2*, *NACC2*, *LFNG* and *HNF1B*, marginally reversed resistance and knockdown of FHL or MAF further enhanced resistance (Supplementary Fig. 7), implying that miR-200c may target multiple genes and pathways with distinct roles in resistance. Meanwhile, we also screened the 16 miR-200c targets for regulation of EMT by performing western blotting for E-cadherin and Vimentin (Fig. 6g). Only knockdown of NOTCH1 partially reversed EMT by increasing E-cadherin without affecting Vimentin, an effect similar to that of miR-200c (Fig. 6d), further suggesting that NOTCH1 promotes EMT to mediate ZEB1-induced resistance.

**Figure 7:**
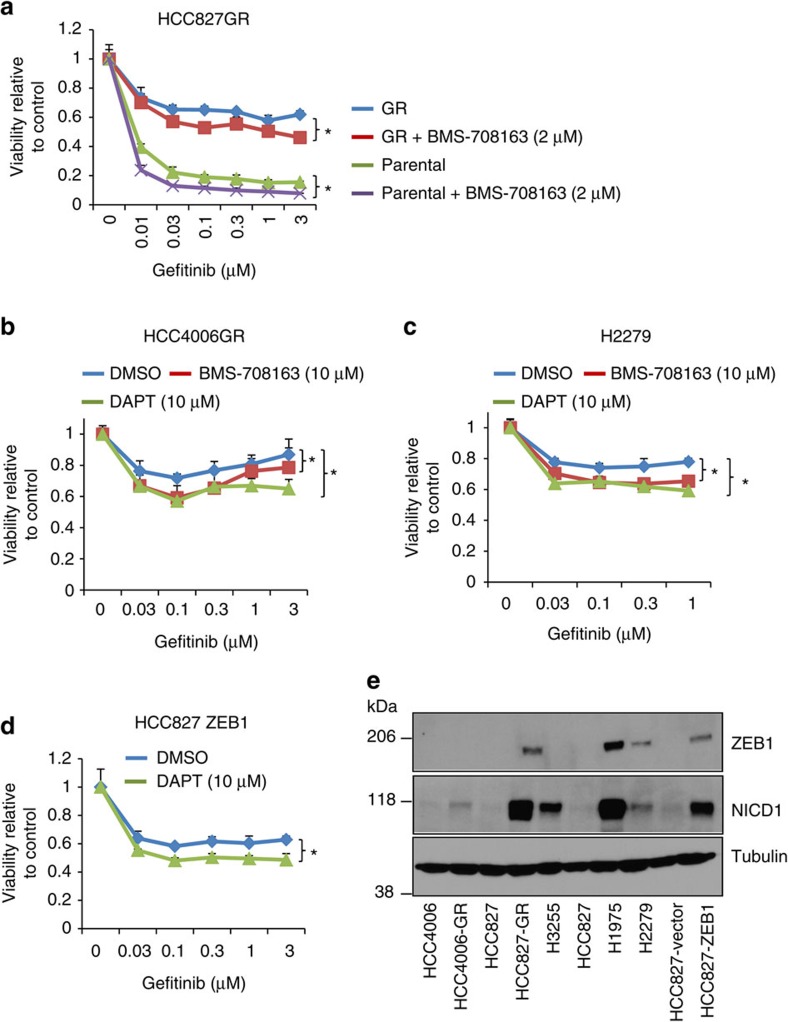
NOTCH1 promotes gefitinib resistance. (**a**) MTT assay for parental HCC827 and HCC827GR cells treated with or without gefitinib and in the presence or absence of BMS-708163 as indicated (triplicates; mean plus s.d.). (**b**–**d**) MTT assays for multiple gefitinib-resistant cell lines treated with or without γ-secretase inhibitors, including BMS-708163 or DAPT as indicated (triplicates; mean plus s.d.). * in the above figures indicates one-way ANOVA test, *P*<0.05. (**e**) Western blotting of ZEB1, NICD1 and Tubulin for lung cancer cell lines that are sensitive or resistant to gefitinib.

To examine the effect of pharmacologic inhibition of NOTCH in regulation of cell growth, we treated the gefitinib-resistant HCC827GR cells with the γ-secretase inhibitor BMS-708163, which dose dependently inhibited NICD1 expression (Fig. 6h), increased the expression of ERBB3 (Fig. 6h) and promoted cell growth (Fig. 6i). These results were consistent with NOTCH1 inhibiting both ERBB3 and cell growth (Figs 3, 4, 5). However, both the parental HCC827 and HCC827GR cells became more sensitive to gefitinib in the presence of BMS-708163 (Fig. 7a), suggesting that the combination of EGFR and NOTCH inhibitors may be useful for treating EGFR inhibitor resistance. In support of this idea, treating multiple gefitinib-resistant lung cancer cells (including HCC4006GR, H2279 and HCC827-ZEB1) with BMS-708163 or DAPT (another γ-secretase inhibitor) also reversed their resistance phenotype to various extents (Fig. 7b–d), demonstrating that NOTCH activation exerts a universal biologic function in EGFR inhibitor resistance in lung cancer cells, and that NOTCH inhibitors may have a therapeutic value in treating EGFR inhibitor-resistant lung tumours.

It is known that distinct mechanisms can drive the acquired resistance to EGFR inhibitors. For instance, HCC4006 cells acquire resistance through loss of EGFR copy number^29^ and HCC827 cells could develop resistance through gefitinib-resistant EGFR T790M mutation-dependent and -independent mechanisms^29^,^30^. To determine whether changes in EGFR mutation effect ZEB1 induction and NOTCH signalling, we examined the expression levels of ZEB1 and NICD1 in multiple gefitinib-resistant cell lines, including HCC827-GR, HCC4006-GR, HCC827-ZEB1, H1975 and H2279 (Fig. 7e and Supplementary Fig. 8). Gefitinib-resistant HCC827-GR and HCC4006-GR cells were generated by continuously treating HCC827 and HCC4006 cells with 1 μM gefitinib for 10–12 weeks until the cells could be grown into confluence and passaged normally. H1975 is known to harbour T790M *EGFR* mutation and sequencing of the genomic DNA from gefitinib-resistant cell lines revealed that only H1975 cells had T790M mutation (Supplementary Fig. 9). HCC827-ZEB1 cells were stable transfectants of the parental HCC827 cells and RNA sequencing experiment (partly shown in Supplementary Fig. 4 and Supplementary Data 1) showed that there was no T790M mutation in these cells. Compared with gefitinib-sensitive parental cells (HCC827, HCC4006 and H3255) or control cells (HCC827 vector), all gefitinib-resistant lines had higher levels of ZEB1 except for HCC4006-GR cells (Fig. 7e) and all gefitinib-resistant lines also expressed higher NICD1 except for H2279 cells (Fig. 7e), suggesting that changes in EGFR mutation may not affect ZEB1 induction or NOTCH1 signalling.

## Discussion

EMT has been an active area of investigation in cancer biology. Its best-characterized role involves the later stage of solid tumour development, when it acts to centrally initiate tumour cell dissemination by destroying the polarized epithelial cell structure and by remodelling the cytoskeleton to mobilize cells^18^,^19^,^20^. Consistently, several families of E-box-binding transcription factors, including the ZEB factors, are known to promote invasion and metastasis by inducing EMT in various cancer cell types^18^,^19^,^20^. Furthermore, inactivation of these factors blocks EMT and suppresses invasion and metastasis in models of metastatic cancer^5^,^6^,^31^,^32^,^33^,^34^, suggesting that these factors and their associated pathways may have therapeutic value. As such, our findings that ZEB1 distinctly regulates (promotes or suppresses) the growth of lung cancer cells harbouring *KRAS* or *EGFR* mutations (Fig. 2) is an important new observation. These findings not only reveal that ZEB1 can act as either an oncogene or a growth suppressor, but also suggest that targeting ZEB1 may have opposite outcomes. The distinct effects of ZEB1 were independent of EMT, indicating that both EMT-dependent and EMT-independent mechanisms collectively mediate the biologic functions of ZEB1 and the latter may specifically mediate the growth suppressive action of ZEB1 in *EGFR*-mutated cells.

ERBB3 belongs to the EGFR family of receptor tyrosine kinases, which includes EGFR and ERBB2-4. Unlike other members of this family, ERBB3 does not have an active kinase domain; it forms heterodimers with EGFR or ERBB2/4 to activate downstream signalling pathways, such as the RAS/mitogen-activated protein kinase pathway^35^,^36^,^37^. Our results identify multiple miR-200c targets as novel mediators of ZEB1 that antagonize ERBB3 and suppress growth, probably through both direct and indirect mechanisms (Figs 3, 4, 5). For instance, TOB1 was reported to bind and to inhibit ERBB2 (refs 38, 39), suggesting a possible indirect mechanism whereby TOB1 suppressed ERBB3 phosphorylation through inhibition of ERBB2. Of particular interest, we found that NOTCH1 directly suppressed *ERBB3* transcription, thereby establishing a molecular link between ZEB1 and ERBB3 repression. As depletion of NOTCH1 promotes ERBB3 in cells harbouring various gene mutations, such a NOTCH1–ERBB3 axis may be widely present in lung cancer cells and serve as a common signalling mechanism that regulates the growth of ERBB3-dependent cells, especially *EGFR*-mutated cells. However, in *KRAS*-mutated cells constitutive KRAS activation may bypass ERBB3 to promote growth, allowing the cells to escape growth inhibition induced by ZEB1 through NOTCH1-mediated *ERBB3* repression. This may also partly explain why ZEB1 distinctly regulates the growth of cells harbouring mutant *KRAS* or *EGFR*.

RBPJ plays a critical role in NOTCH1-mediated transcription regulation^27^. In the absence of NICD1, RBPJ can recruit transcription co-repressors to silence gene expression. When NICD1 is activated and translocates into the nucleus, it can promote target gene transcription by binding to RBPJ, recruiting co-activator MAML1 and converting the initially transcription-inactive RBPJ complexes into activators^27^. Although our results suggest that NICD1 binds to both RBPJ and *ERBB3* promoter, it remains unclear whether NICD1 directly represses *ERBB3* transcription through binding to RBPJ. It should be noted that our current results can not preclude the possibility that NICD1 may also repress ERBB3 through other indirect mechanisms, for example, through HES or HEY family of transcription repressors, both of which are well-established NICD1 transcription targets^27^. Thus, future studies are needed to determine whether multiple mechanisms are involved in NICD1-mediated ERBB3 repression.

Previous studies have shown that ZEB1 promoted the NOTCH ligand Jagged1 expression and activated NOTCH signalling in pancreatic and prostate cancer cells by repressing miR-200 family members, which directly targeted the 3′-UTR of Jagged1 (refs 40, 41). Our results showed that ZEB1 promoted NOTCH1 but not Jagged1 in lung cancer cells (Supplementary Fig. 4 and Supplementary Data 1), suggesting that the ZEB1/miR-200 axis may regulate NOTCH signalling through differential mechanisms in different types of human cancer cells.

Tyrosine kinase inhibitors of EGFR, including gefitinib and erlotinib, are clinically effective drugs that treat *EGFR*-mutated lung cancer. However, their efficacy is transient and drug resistance inevitably develops, leaving patients with few treatment options. How resistance is generated remains incompletely understood. Several genetic mechanisms, including drug-resistant *EGFR* mutations and *MET* gene amplification, drive resistance in more than half of the affected patients^28^,^42^; these patients may be treatable with newer-generation EGFR inhibitors or MET inhibitors, which are in clinical development. However, for patients without these genetic changes, the mechanisms of resistance remain elusive and effective targeted therapeutics do not exist. Intriguingly, EMT drives resistance in a subset of these patients^28^,^42^,^43^. As such, our results that ZEB1 promoted EMT and resistance partially through repression of miR-200c (Fig. 6) are exciting. These results are consistent with recent reports from others showing that miR-200 inhibits EMT and chemoresistance in breast tumour cells, and that EMT inhibition also promotes sensitivity to gemcitabine treatment in pancreatic cancer cells^44^,^45^. Mechanistically, our data show that NOTCH1 is a direct target of miR-200c and drives EMT and resistance (Figs 5, 6), thereby providing a novel and direct molecular link between miR-200 and the regulation of these processes. Further, our studies show that NOTCH1 depletion or treatment with γ-secretase inhibitors reverse resistance in multiple gefitinib-resistant lung cancer cells (Figs 6, 7), suggesting a strong translational potential of our above mechanistic findings.

Notably, our results show that treatment with γ-secretase inhibitors (BMS-708163 and DAPT) partially reversed the resistance phenotype (Fig. 7), an effect similar to NOTCH1 knockdown or miR-200c expression, suggesting that additional pathways may also be implicated in ZEB1-induced EMT and resistance. In addition, our data are consistent with recent reports^46^,^47^,^48^ showing that ZEB1 or NOTCH1 promotes EMT and EGFR inhibitor resistance in lung cancer cell lines, including HCC4006, PC9 and H1650. Taken together, these findings suggest that NOTCH1 antagonists, with combined use of EGFR inhibitors, may be useful for treating patients with EMT-dependent resistance. Furthermore, a recent report has shown that HER2 inactivation led to the activation of NOTCH1, which in turn promoted mammary tumour dormancy and recurrence^49^, suggesting that NOTCH1 inhibition may have broader applications in the treatment of drug resistance in multiple human cancers.

Nevertheless, it should be noted that the inhibition of NOTCH1 by γ-secretase inhibitor treatment or by siRNAs also significantly promoted both ERBB3 expression and lung cancer cell growth in the absence of gefitinib (Figs 3, 4, 5, 6). This is different from the observation from breast cancer cells that NOTCH1 inhibition reduced tumour cell growth^49^. Our findings indicate that the inactivation of NOTCH1 alone, without concomitant inhibition of EGFR, may lead to unexpected outcomes in lung cancer by increasing ERBB3 to promote *EGFR*-mutated tumour growth, providing a caution against indiscriminate use of NOTCH inhibitors. Thus, personalized therapeutics should be developed to direct the use of these drugs in the treatment of *EGFR*-mutated lung cancer.

## Methods

### Antibodies and reagents

Rabbit monoclonal anti-ZEB1 (3396, s; clone D80D3), cleaved NOTCH1 (4147, s; clone D3B8; specific for detecting NICD1), RBPJ (5313, s; clone D10A4), E-cadherin (3195, s; clone 24E10), Vimentin (5741, s; clone D21H3), Tubulin (2125, s; clone 11H10), pERBB3 (2842, s; clone D1B5) and ERBB3 (12708, s; clone D22C5) were purchased from Cell Signaling and used at a 1:3,000 dilution for western blotting. Goat polyclonal anti-Actin (sc-1616) was purchased from Santa Cruz and used at a 1:1,000 dilution for western blotting. Rabbit polyclonal anti-NOTCH1 (ab27526) was purchased from Abcam and used at a 1:3,000 dilution for western blotting. Mouse monoclonal anti-FLAG (F1804; monoclonal; clone M2) was purchased from Sigma and used at a 1:5,000 dilution for western blotting. siRNAs were purchased from Santa Cruz (pooled siRNAs) or Origene (individual siRNAs for ZEB1 and RBPJ). All short hairpin RNAs and chemicals were purchased from Sigma, except for BMS-708163 and gefitinib, which were purchased from Selleckchem. Control GFP (LPP-EGFP-LV105-025) and ZEB1 lentiviral particles (LPP-F0876-Lv105-200-S) were purchased from Genecopoiea.

### Cell culture and transfection

Mycoplasma-free and authenticated (short tandem repeat (STR) profiling) human lung epithelial cell line BEAS2B and lung adenocarcinoma cell line HCC4006 were purchased from American Type Culture Collection. All other lung adenocarcinoma cell lines (including HCC827, H3255, H1975, H2279, H322. 393P, H441 and H1299 cells) were gifts from Jonathan Kurie M.D. (MD Anderson Cancer Center). All cells were cultured in RPMI-1640 (Mediatech) supplemented with 10% fetal bovine serum (Life Technologies). Lipofectamine 2000 and RNAiMAX (both from Life Technologies) were used for transfection of DNA or RNA, respectively. For stable transfection, antibiotic selection was introduced at 48 h after transfection. To generate gefitinib-resistant sublines (HCC827GR and HCC4006GR), HCC827 and HCC4006 cells were continuously treated with 1 μM gefitinib for 10–12 weeks.

### DNA sequencing

Genomic DNA was extracted using the QIAamp DNA Mini Kit (Qiagen). Genomic exons 18–21 of the *EGFR* gene and exons 2–4 of the *K-RAS* gene were separately amplified using AmpliTaq Gold 360 PCR Master Mix (Life Technologies). The PCR was performed with a denaturing step at 95 °C for 5 min, then 30 s at 95 °C, 30 s at 56 °C and 30 s at 72 °C for 35 cycles, followed by a final 7 min at 72 °C. The PCR products were visualized on a 1% agarose gel and then subjected to direct sequencing within Mayo Clinic Molecular Biology core. This following sequencing primers were used: EGFR exon 18: 5′-CTGAGGTGACCCTTGTCTCTG-3′ EGFR exon 19: 5′-TGCCAGTTAA- CGTCTTCCTT-3′ EGFR exon 20: 5′-CATTCATGCGTCTTCACCTG-3′ EGFR exon 21: 5′-TGATCTGTCCCTCACAGCAG-3′ EGFR T790M: 5′-CTCCAGGAAGCCTACGTGAT-3′ KRAS exon 2: 5′-AAGGCCTGCTGAAAATGACTG-3′ KRAS exon 3: 5′-GCACTGTAATAATCCAGACT-3′ KRAS exon 4: 5′-GACAAAAGTTGTGGACAGGT-3′.

### Constructs

3 × FlagNICD1 (plasmid #20183, a gift from Raphael Kopan, Cincinnati Children's Hospital^50^) and ErbB-3-pGL3 (plasmid #60899, a gift from Frederick Domann, The University of Iowa^51^) were from Addgene. Mouse ZEB1 complementary DNA was cloned into pcDNA3.1 vector (cloning primers: 5′-TCGAATTCATGGCGGATGGCCCCAGGTGTAA-3′ and 5′-GAGCGGCCGCCTAAGCTTCATTTGTCTTCTCTT-3′). Full-length 3′-UTRs of TOB1, RANBP9, LFNG, NOTCH1, BCL2, FHL and KLF9 were cloned from the HCC827-ZEB1 cells by PCR and inserted into the pCI-hRL vector. The Q5 Site-Directed Mutagenesis Kit (NEB) was used to generate mutant constructs. The following primers were used to clone the wild-type or mutant 3′-UTR constructs for the above genes:

TOB1 3′-UTR: 5′-GCTCTAGATTCTAACCAGCAATTCCAGC-3′ (forward) and 5′-TTGCGGCCGCAACTATTTCAGTCCCTCTT-3′ (reverse); miR-200c-binding site mutant TOB1 3′-UTR: 5′-CTTTAAGATATAGCAAGGACATG-3′ (forward) and 5′-GGTGTAAGAGGCCATATCTGAG-3′ (reverse).

RANBP9 3′-UTR: 5′-GCTCTAGACCACAGTGGAAGACTACCTA-3′ (forward) and 5′-TTGCGGCCGCAGAAGTAAATTTTTAATGGC-3′ (reverse); miR-200c-binding site mutant RANBP9 3′-UTR: 5′-CTTACATGTCAACGGTGTGGTTATG-3′ (forward) and 5′-GGTGTTATAATACAACAGTTAAACTTGTGAGTC-3′ (reverse).

LFNG 3′-UTR: 5′-GCTCTAGATTCTAGTGGCCATGGCTGAG-3′ (forward) and 5′-TTGCGGCCGCGCTGCAAAGAGCACCTTT-3′ (reverse); miR-200c-binding site mutant LFNG 3′-UTR: 5′-CTTTTTTTACTGTGCTGTTTTTTTTG-3′ (forward) and 5′-GGTGTCACAATATTTACAGACACG-3′ (reverse).

NOTCH1 3′-UTR: 5′-GCTCTAGAGCCGACCAGAGGAGCCTTTT-3′ (forward) and 5′-TTGCGGCCGCAACATCTTGGGACGCATCTGG-3′ (reverse); miR-200c-binding site mutant NOTCH1 3′-UTR: 5′-CTTATGTAGTTGTTCGTTGGTTATAC-3′ (forward) and 5′-GGTGAACCTGAAACAAAGATTCATG-3′ (reverse).

BCL2 3′-UTR: 5′-GCTCTAGAGCCACTGAGGAGCTTTGTTT-3′ (forward) and 5′-TTGCGGCCGCGGCCTCTCTTGCGGAGTATT-3′ (reverse).

FHL 3′-UTR: 5′-GCTCTAGAATCTGGCCAACAAGCGCTTT-3′ (forward) and 5′-TTGCGGCCGCGCAGGTTATTCATATGCTGC-3′ (reverse).

KLF9 3′-UTR: 5′-GCTCTAGACTGCACGCTGCCTTTTAGTG-3′ (forward) and 5′-TTGCGGCCGCTGATTTACAAAAACGGGACAGCA-3′ (reverse).

### Quantitative RT–PCR

Cells were lysed in Trizol reagent (Life Technologies) and total RNA was extracted with the Qiagen RNAeasy mini kit. Quantitative reverse transcriptase–PCRs (RT–PCRs) for mRNAs and microRNAs were performed using the respective kits from Applied Biosystems (Superscript III for mRNAs and TaqMan assays for microRNAs). The following primers were used for the quantitative RT–PCRs:

Human ZEB1: 5′-CACTGGTGGTGGCCCATTAC-3′ (forward) and 5′-TGCACCATGCCCTGAGG-3′ (reverse).

Human CDH1: 5′-GACACACCCCCTGTTGGTGT-3′ (forward) and 5′-CAGCCATCCTGTTTCTCTTTCAA-3′ (reverse).

Human VIM: 5′-GGAACAGCATGTCCAAATCGA-3′ (forward) and 5′-GCCGTGAGGTCAGGCTTG-3′ (reverse).

Human L32: 5′-CCTTGTGAAGCCCAAGATCG-3′ (forward) and 5′-TGCCGGATGAACTTCTTGGT-3′ (reverse).

Human RBPJ: 5′-GTGCTGGATCTGGGAATCTCT-3′ (forward) and 5′-GGTTTTAGGACGCGCTTTGA-3′ (reverse).

Human ERBB3: 5′-ACATCGTGAGGGACCGAGA-3′ (forward) and 5′-GGACAGCTTCTGCCATTGTC-3′ (reverse).

Human NOTCH1: 5′-ATCCTGATCCGGAACCGAG-3′ (forward) and 5′-CGTCGTGCCATCATGCAT-3′ (reverse).

Human TOB1: 5′-CACTAACGGCGATCTCCCAA-3′ (forward) and 5′-TGAGGACAGAGGACAGAGGC-3′ (reverse).

Human RANBP9: 5′-GCCCGAAGGACAAGTTCAGC-3′ (forward) and 5′-CCGCAGGTTGTTCTGAGAGAG-3′ (reverse).

Human LFNG: 5′-CCACCAAAAAGTTCCACCGC-3′ (forward) and 5′-CGAGATCCAGGTCTCCAGCA-3′ (reverse).

Human SURF4: 5′-ATGGGCCAGAACGACCTGAT-3′ (forward) and 5′-GAGGAACTGGTCGGCGAAG-3′ (reverse).

Human MAF: 5′-CCCGAGTTTTTCATAACTGAGCC-3′ (forward) and 5′-CCCACTGATGGCTCCAACTT-3′ (reverse).

Human FHL1: 5′-TATCTGCCACACATCCAGCG-3′ (forward) and 5′-CCATGGTGCCCACCTTGTAG-3′ (reverse).

Human FLI1: 5′-TATCTGCCACACATCCAGCG-3′ (forward) and 5′-ACGCTGAGTCAAAGAGGGAC-3′ (reverse).

Human NACC2: 5′-AAAGCGAACAGGGAAACCGA-3′ (forward) and 5′-TCCACTGTCTGAAACGGCTC-3′ (reverse).

Human HEG1: 5′-AACGTTCGATCGCTGGGATT-3′ (forward) and 5′-TTCAATAGCTGTGCCACGCA-3′ (reverse).

Human HNF1B: 5′-TGGTACGTCAGAAAGCAACGA-3′ (forward) and 5′-GAACTCTGGACTGTCTGGTTGA-3′ (reverse).

Human PRKAR2B: 5′-AGTTGCCCTGTTTGGAACGA-3′ (forward) and 5′-TGCTTCATGCAGTGGGTTCA-3′ (reverse).

Human GLI3: 5′-CGGGTCTATGGGAAGTTCGG-3′ (forward) and 5′-GACCAAAAATGCCCTGCGG-3′ (reverse).

Human BCL2: 5′-TGGGATGCCTTTGTGGAACT-3′ (forward) and 5′-GAAATCAAACAGAGGCCGCA-3′ (reverse).

Human NR5A2: 5′-TTACACCTATTGGTGCTGGGC-3′ (forward) and 5′-GGGGATGGGGGATCCGT-3′ (reverse).

Human KLF9: 5′-CAACACTCGGTCCCCTTGAA-3′ (forward) and 5′-CTCCAACAGTCAGAGACGGG-3′ (reverse).

Mouse ZEB1: 5′-ATGCTCTGAACGCGCAGC-3′ (forward) and 5′-AATCGGCGATCTTTGAGAGCT-3′ (reverse).

Mouse CDH1: 5′-ACTGCGCTGGATAGTGTGTG-3′ (forward) and 5′-GTGGAGAGGGAATACCACGA-3′ (reverse).

Mouse VIM: 5′-TCCAAGCCTGACCTCACTGC-3′ (forward) and 5′-TTCATACTGCTGGCGCACAT-3′ (reverse).

Mouse L32: 5′-GGAGAAGGTTCAAGGGCCAG-3′ (forward) and 5′-TGCTCCCATAACCGATGTTTG-3′ (reverse).

### MTT assay

Briefly, cells were seeded on a 96-well plate (2,000 cells per well) and cultured for 1–5 days. Cells were then incubated with MTT (3-(4,5-dimethylthiazol-2-yl)-2,5-diphenyltetrazolium bromide; 1 mg ml^−1^) for 3 h and cell growth was quantified by measuring absorbance at 570 nm.

### Phospho-receptor tyrosine kinase array

The human receptor tyrosine kinase (RTK) array was performed by using a kit purchased from R&D (ARY001B), which has antibodies spotted in duplicate, to detect the tyrosine phosphorylation of 49 different RTKs. Briefly, 80–90% confluent HCC827 vector and HCC827-ZEB1 cells were lysed in lysis buffer provided by the kit and protein lysates were prepared exactly as instructed in the product manual. The array films were sequentially incubated with fresh protein lysates and anti-phospho-tyrosine antibody (provided by the kit). Phosphorylated-RTK spots were then visualized by incubating with ChemiReagent provided by the kit as instructed by the product manual.

### Western blotting

Whole-cell protein extraction was performed by lysing in RIPA lysis buffer system (Santa Cruz). The extracted proteins (10–30 μg) were separated by SDS–PAGE electrophoresis, transferred onto polyvinylidene difluoride membranes, blocked with 5% skim milk and incubated with primary antibodies overnight. Protein bands were visualized by incubating with an horseradish peroxidase-conjugated secondary antibody and supersignal ECL substrates (Pierce). Uncut western blotting films were included in Supplementary Fig. 10.

### RNA sequencing

Total RNA was prepared using the miRNeasy kit (Qiagen). RNA sequencing was performed by Mayo Clinic gene expression core laboratory. The sequencing data have been deposited to the GEO repository with an accession code GSE81167 (www.ncbi.nlm.nih.gov/geo/query/acc.cgi?acc=GSE81167).

### Reporter assay

Cells were transfected with reporter plasmids using Lipofectamine 2000. After 48 h, cells were lysed in passive lysis buffer (Promega) and luciferase activity was measured using a Dual-Luciferase Reporter Assay System (Promega).

### Human and mouse studies

Human tissues were acquired from Mayo Clinic lung tissue registry with protocols approved by the Mayo Clinic internal review board and human tissue subcommittee. Mice were purchased from Charles Rivers. All protocols were approved by the Mayo Clinic Institutional Animal Care and Use Committee (IACUC). Briefly, cells (one million per mouse) were suspended in PBS and subcutaneously injected into the flanks of 8- to 10-week-old male athymic nude mice (strain 490; *n*=6 per cohort for HCC827 vector and HCC827-ZEB1 xenograft tumours; *n*=10 per cohort for H1975-sc and H1975-TOB1sh4 xenograft tumours) or male wild-type 129S2/SvPasCrl mice (strain 476; *n*=10 per cohort for 393P-vector and 393P-ZEB1 xenograft tumours). Autopsies were performed at 3 weeks after injection.

### Statistics

Statistical significance was determined using two-sided Student's *t*-tests or one-way analysis of variance as indicated, with *P*<0.05 being considered a statistically significant difference, unless otherwise indicated.

### ChIP assays

ChIP assay was performed using ChIP-IT Express Enzymatic Kit (Active Motif). Briefly, cells were cross-linked with 1% formaldehyde and then lysed. After enzymatic digestion, samples were immunoprecipitated with anti-NOTCH1 (Abcam 27526; 1:200 dilution) or anti-RBPJ (Cell Signaling 5313, s; 1:200 dilution), or control normal anti-Rabbit IgG (Cell Signaling Technology). DNA was eluted and quantitative PCR was carried out with specific primers to amplify the RBPJ-binding site of the ERBB3 promoter (forward: 5′-GACGGTGCGGCCAGACTCCA-3′ and reverse: 5′-TCTCCCGGGGATTTGGAT-3′).

### Immunohistochemistry

For immunohistochemistry, the frozen samples were cut into 10-μm sections using a cryostat microtome (Leica). The slides were fixed in cold menthol for 10 min at −20 °C and washed with tris-buffered saline (TBS). Subsequently, the sections were incubated in 3% H_2_O_2_ solution in methanol at room temperature for 10 min. After washing, blocking buffer (5% normal goat serum/tris-buffered saline (TBS)) was added and incubated for 30 min at room temperature. The sections were then incubated with primary antibodies 4 °C overnight (anti-ZEB1: 1:100 dilution; anti-E-cadherin: 1:400 dilution; and anti-Vimentin: 1:400 dilution). The detection system used was the avidin–biotin complex method. Finally, the sections were rinsed, counterstained with haematoxylin and mounted on glass slides before evaluation. The slides were examined by a pathologist using light microscopy.

### Data availability

RNA sequencing data have been deposited to the GEO repository (accession code GSE81167) and relevant data are available from the authors.

## Additional information

**How to cite this article**: Zhang, T. *et al*. A genetic cell context-dependent role for ZEB1 in lung cancer. *Nat. Commun.* 7:12231 doi: 10.1038/ncomms12231 (2016).

## Supplementary Material

Supplementary Information Supplementary Figures 1-10 and Supplementary Tables 1-4

Supplementary Data 1 RNA sequencing of HCC827 cells expressing ZEB1 or empty pcDNA3.1 vector (3.1)

## Figures and Tables

**Table 1:** Lung adenocarcinoma/normal lung fold changes of EMT-related genes in the Oncomine data sets.

Genes	Data set	Beer	Bhattacharjee	Selamet	Hou	Garber	Stearman	Landi	Su	Okayama
*CDH1*	Fold change	**1.851**	1.301	**2.586**	**1.444**	**1.636**	**2.595**	**2.24**	**2.152**	**1.928**
	*P*-value	**2.74e−10**	0.253	**4.1e−20**	**1.38e−5**	**0.005**	**7.43e−9**	**5.59e−19**	**7.12e−6**	**1.88e−12**
*VIM*	Fold change	**−1.943**	**−3.3739**	**−2.664**	**−2.781**	**−1.586**	**−1.383**	**−1.634**	**−1.71**	**−1.446**
	*P*-value	**1.13e−13**	**1.23e−4**	**3.36e−27**	**6.03e−13**	**0.001**	**4.03e−5**	**4.84e−14**	**1.59e−5**	**0.001**
*ZEB1*	Fold change	NA	NA	**−1.15**	**−2.652**	−1.363	NA	**−1.583**	**−2.814**	**−2.529**
	*P*-value	NA	NA	**1.3e−11**	**15.8e−17**	0.059	NA	**1.67e−8**	**6.08e−7**	**1.5e−9**
*ZEB2*	Fold change	NA	−3.376	−1.131	−1.014	−2.127	−1.719	−2.214	−3.069	1.007
	*P*-value	NA	1	1	0.774	0.995	1	1	1	0.483
*SNAI1*	Fold change	NA	NA	−1.031	−1.093	1.887	NA	−1.023	1.108	−1.369
	*P*-value	NA	NA	0.996	0.889	0.136	NA	0.764	0.309	0.925
*SLUG*	Fold change	NA	1.178	−1.548	1.158	**1.852**	1.037	−1.161	−1.086	−1.885
	*P*-value	NA	0.281	1	0.174	**0.036**	0.449	0.923	0.696	1
*TWIST*	Fold change	**1.735**	1.466	1.062	**2.541**	NA	**3.001**	**1.505**	**1.762**	**1.562**
	*P*-value	**1.12e−4**	0.102	0.055	**3.58e−7**	NA	**2.41e−4**	**5.2e−9**	**0.005**	**0.013**

NA, data not available.

Statistically significant (*t*-test *P*<0.05) changes are highlighted in bold.
