# Supplementary material for: A genetic cell context-dependent role for ZEB1 in lung cancer
Source: Nat Commun. 2016 Jul 26;7:12231. doi: 10.1038/ncomms12231 (PMC4963474; doi:10.1038/ncomms12231)
Supplement: Supplementary Information — Supplementary Figures 1-10 and Supplementary Tables 1-4 [file ncomms12231-s1.pdf]

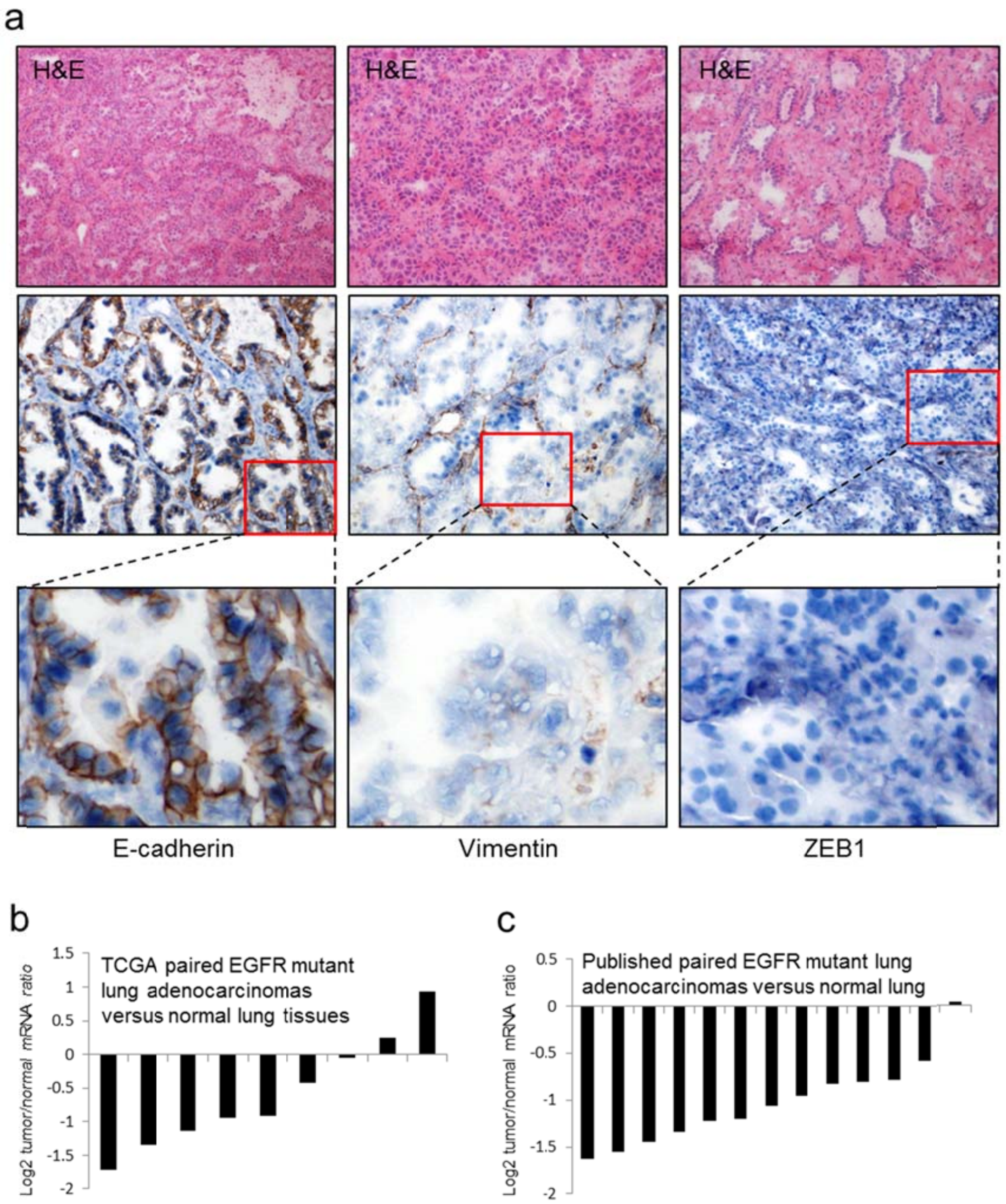

Supplementary Figure 1. ZEB1 is repressed in *EGFR*-mutated lung adenocarcinomas. **(a)** Representative H&E and immunohistochemical staining of frozen sections of lung adenocarcinomas shown in Fig. 1. Magnification: 200X. **(b and c)** The logged (log2) tumour versus normal ratios of *ZEB1* mRNA levels in paired *EGFR*-mutated lung

adenocarcinomas and normal lung tissues from the TCGA dataset (b) or from previous reports (c; references 23 and 24).

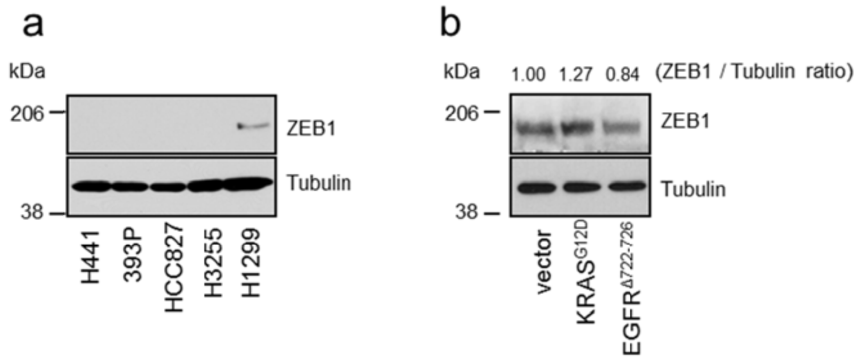

Supplementary Figure 2. Western blotting of ZEB1 and Tubulin for lung cancer cell lines **(a)** or BEAS2B lung epithelial cells transfected with $KRAS^{G12D}$ or $EGFR^{\Delta 722\text{-}726}$ **(b)**. H1299 cells were included in (a) as a positive control. The ZEB1/Tubulin ratio in (b) was determined by Image J software.

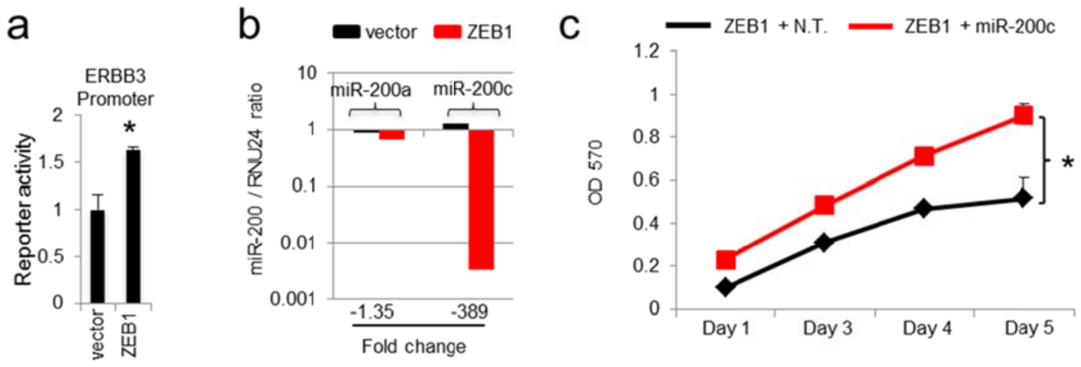

Supplementary Figure 3. ZEB1 does not inhibit ERBB3 promoter and suppresses cell growth by repressing miR-200c. **(a)** ERBB3 promoter reporter assay for H1299 cells co-transfected with ZEB1 cDNA and a luciferase-conjugated ERBB3 promoter reporter plasmid (triplicates; mean plus s.d.). * indicates t-test $p < 0.05$. **(b)** ZEB1 repressed miR-200c in HCC827 cells. The expression of miR-200a and miR-200c was quantitated by qPCR (mean of triplicates) and normalized to that of RNU24 (human internal control for small non-coding RNAs). **(c)** MTT assay for HCC827-ZEB1 cells transiently transfected with miR-200c or a control non-targeting microRNA (N.T.) (triplicates; mean plus s.d.). * indicates t-test $p<0.05$.

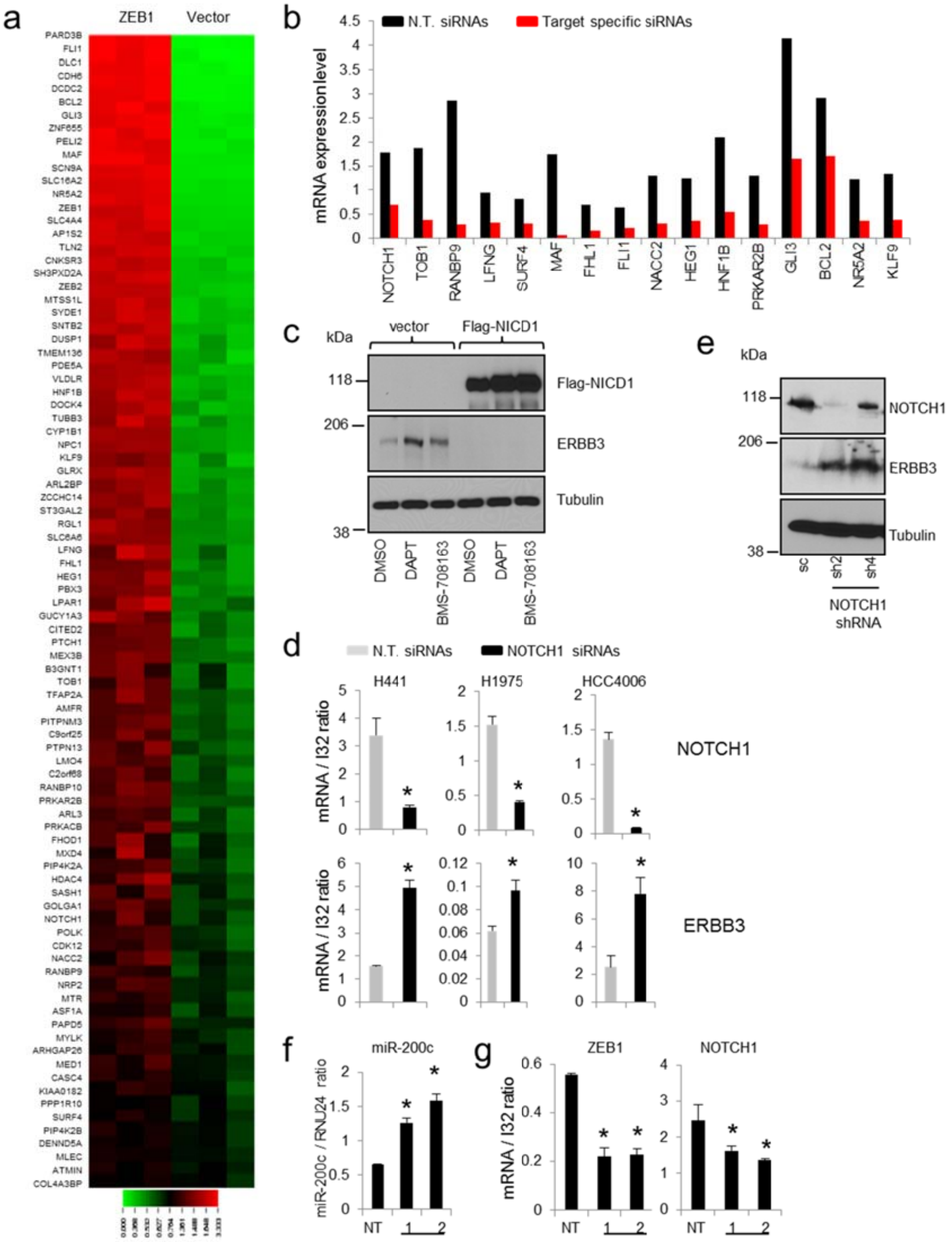

Supplementary Figure 4. ZEB1 decreases miR-200c and increases NOTCH1 to repress ERBB3. **(a)** Heat-map for the 87 predicted miR-200c targets that were increased by

ZEB1 as revealed by RNA-sequencing of the HCC827 cells expressing ZEB1 or pcDNA3.1 vector. **(b)** qPCR for HCC827-ZEB1 cells transiently transfected with siRNAs against each of the 16 randomly selected miR-200c targets (mean of triplicates). **(c)** Western blotting of FLAG, ERBB3, and Tubulin for H1975 cells transfected with an empty vector or FLAG tagged NICD1 (Flag-NICD1) and treated with DMSO or γ-secretase inhibitors DAPT (50 μM) or BMS-708163 (25 μM) overnight. **(d)** qPCR of NOTCH1 and ERBB3 for multiple lung cancer cell lines transiently transfected with NOTCH1 siRNAs (triplicates; mean plus s.d.). * indicates t-test $p < 0.05$. **(e)** Western blotting of cleaved NOTCH1, ERBB3, and Tubulin for HCC827GR cells transfected with NOTCH1 shRNAs or a control scrambled shRNA (sc). **(f)** Knockdown of ZEB1 increased miR-200c in lung cancer cells (H1975). The expression of miR-200c was quantitated by qPCR and normalized to that of RNU24 (human internal control for small non-coding RNAs) (triplicates; mean plus s.d.). * indicates t-test $p < 0.05$. **(g)** Knockdown of ZEB1 decreased NOTCH1 in H1975 cells (triplicates; mean plus s.d.). * indicates t-test $p < 0.05$.

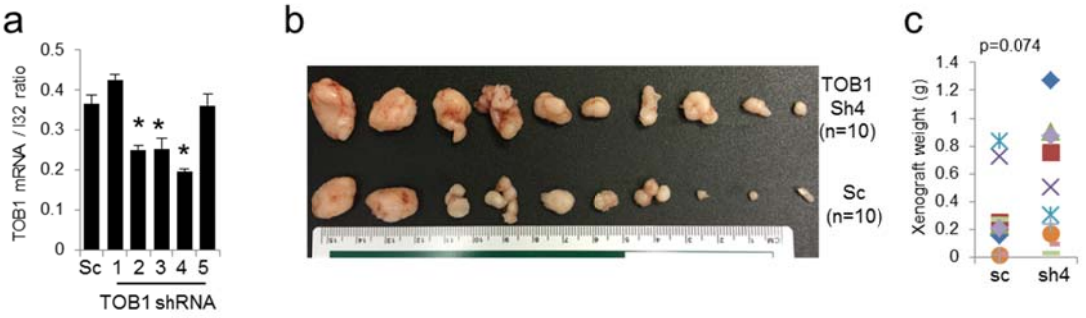

Supplementary Figure 5. The role of TOB1 in xenograft lung tumor growth. **(a)** qPCR for H1975 cells stably expressing TOB1 shRNAs or a control scrambled shRNA (sc) (triplicates; mean plus s.d.). * indicates t-test $p < 0.05$. (**b & c**) Knockdown of TOB1 moderately promoted the subcutaneous xenograft tumour growth of H1975 cells. Tumours were photographed (b) and weighed and measured (c) at three weeks after the subcutaneous injection of the H1975 transfectants (sc & shRNA4).

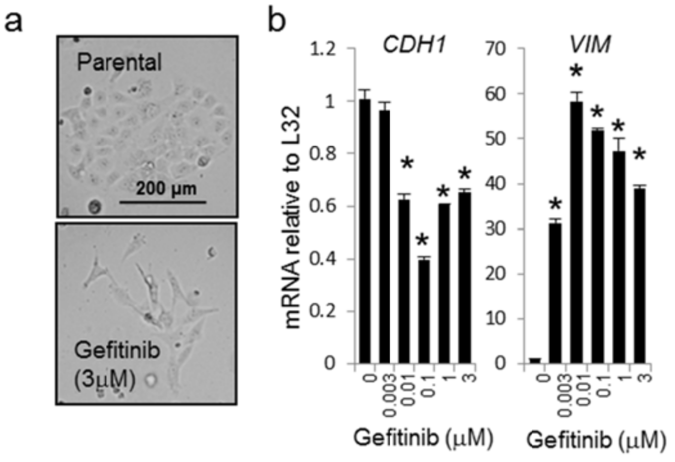

Supplementary Figure 6. gefitinib induces EMT of HCC827 cells. **(a)** Morphology of HCC827 cells treated with or without gefitinib for two weeks. **(b)** qPCR of *CDH1* and *VIM* for HCC827 cells treated with or without gefitinib for two weeks (triplicates; mean plus s.d.). * indicates t-test $p < 0.05$.

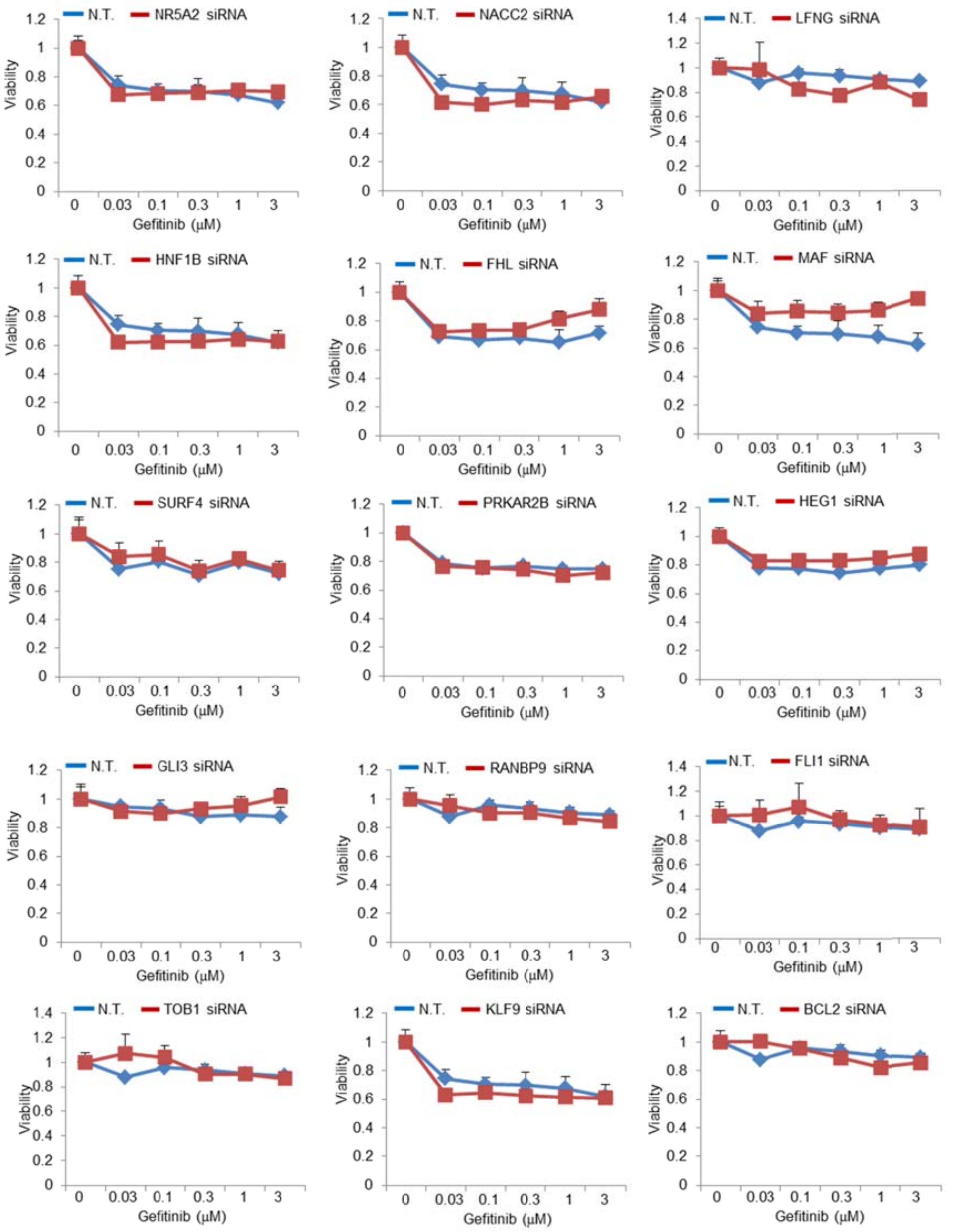

Supplementary Figure 7. The effect of the knockdown of miR-200c targets on gefitinib resistance of HCC827-ZEB1 cells. HCC827-ZEB1 cells were transiently transfected with siRNAs against miR-200c targets or a control non-targeting siRNA (N.T.) and treated

with or without gefitinib. MTT assay was performed three days after gefitinib treatment (quadruplicates; mean plus s.d.).

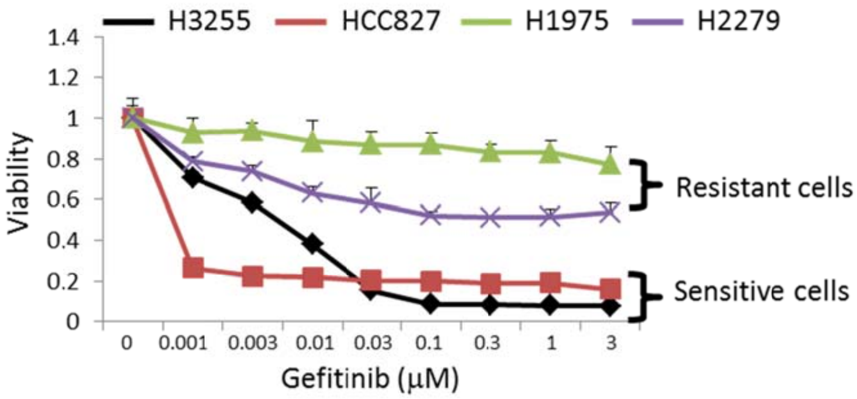

Supplementary Figure 8. The sensitivity of four *EGFR*-mutated lung cancer cell lines to gefitinib treatment. Cells were treated with the indicated concentrations of gefitinib, and MTT assay was performed three days after treatment (quadruplicates; mean plus s.d.).

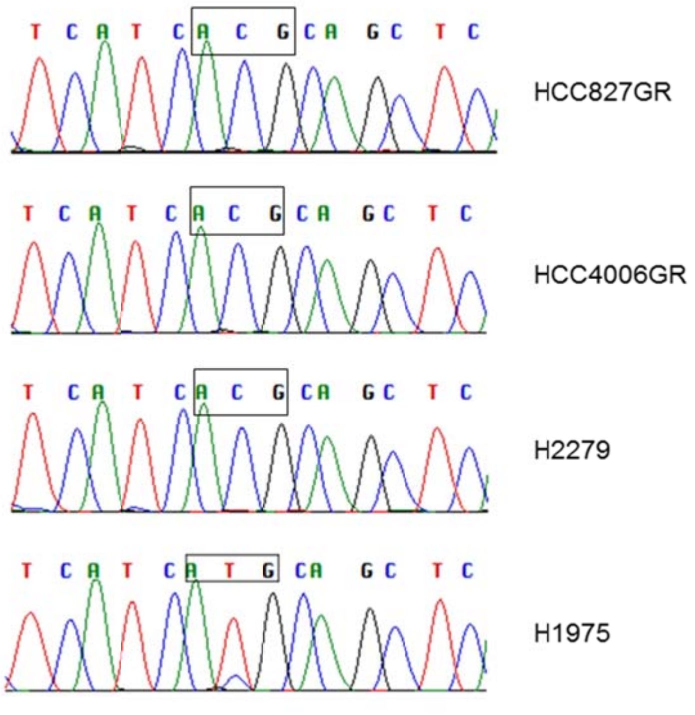

Supplementary Figure 9. The *EGFR* T790M mutation status of gefitinib resistant cell lines. Sequencing of the genomic DNA from four gefitinib resistant cell lines, including HCC827GR, HCC4006GR, H2279, and H1975, showed that only H1975 cells had *EGFR* T790M mutation (ACG to ATG mutation; boxed).

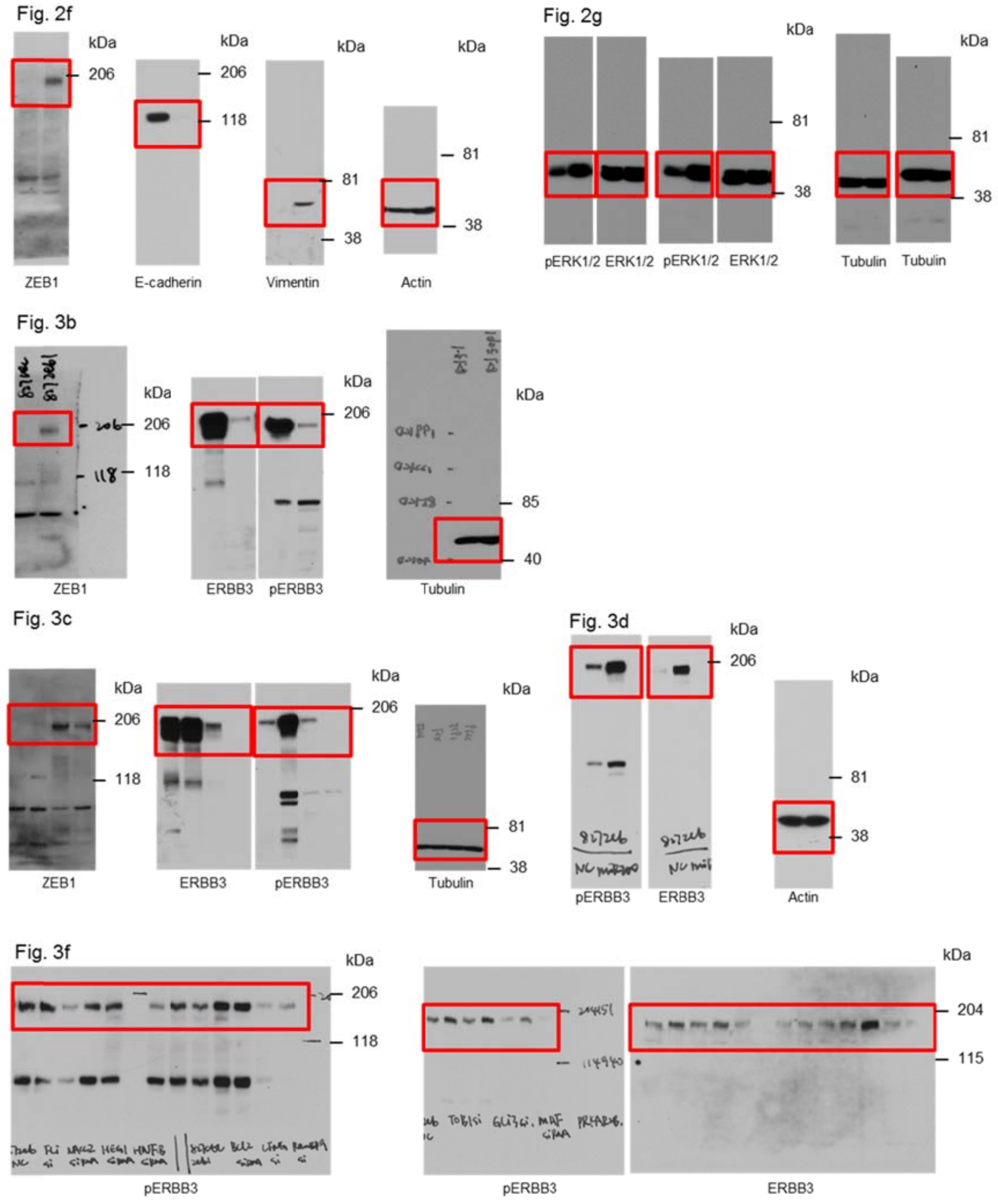

Supplementary Figure 10. Uncut Western blotting films. Red boxed are bands presented in figures.

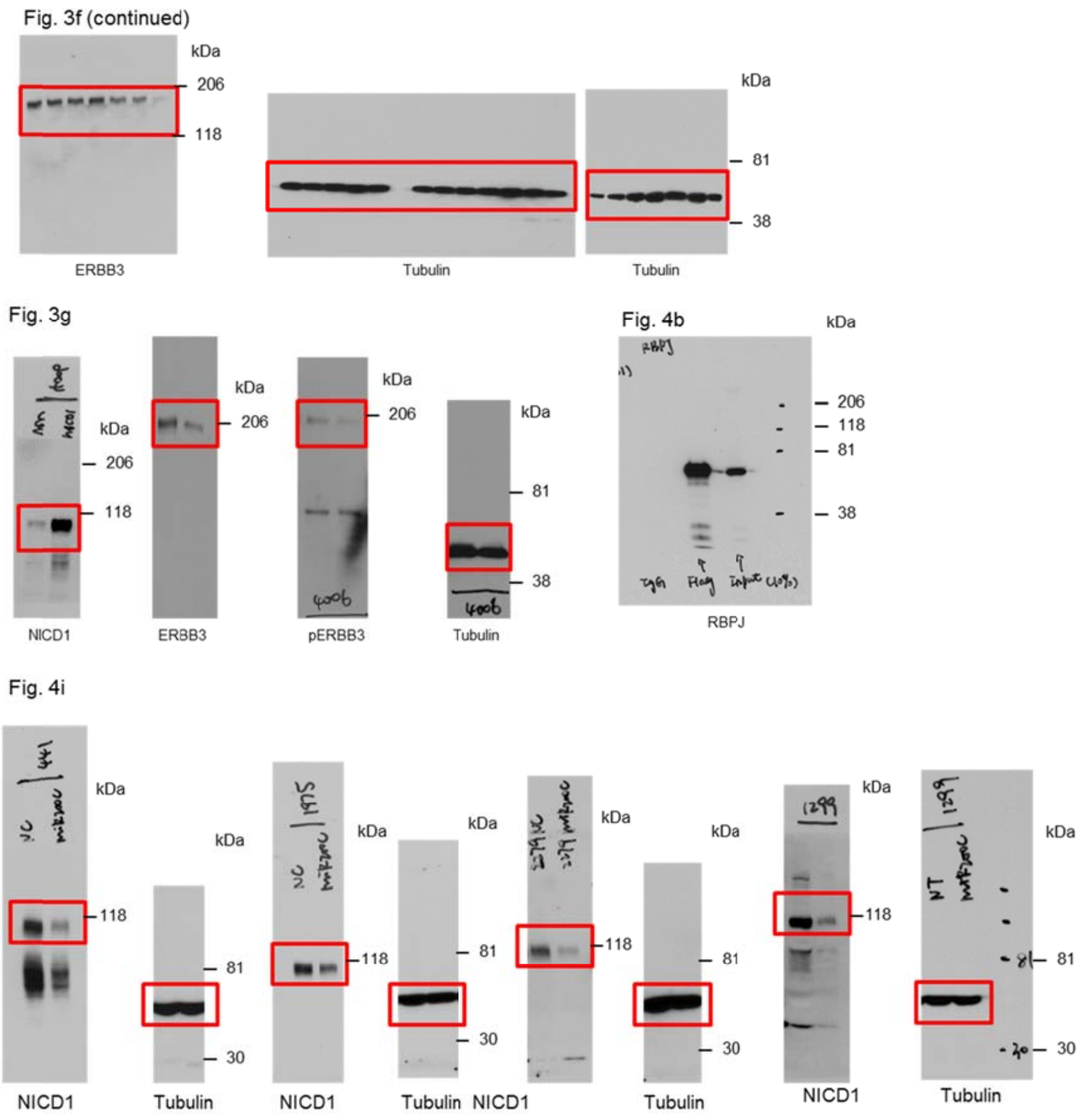

Supplementary Figure 10 (continued). Uncut Western blotting films. Red boxed are bands presented in figures.

Fig. 4j

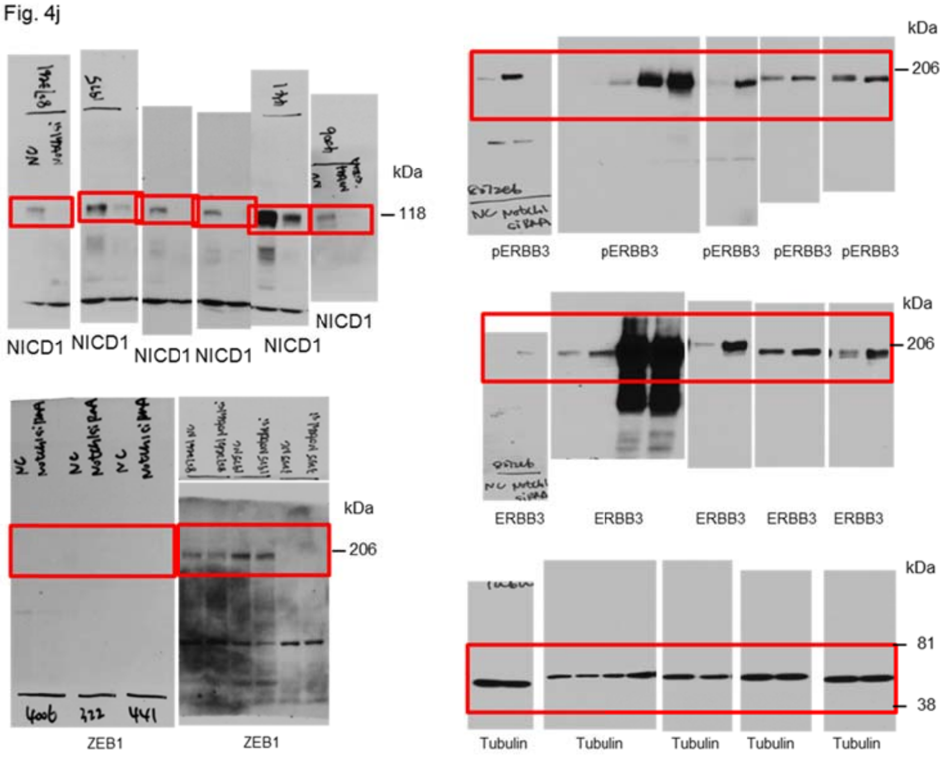

Fig. 6d

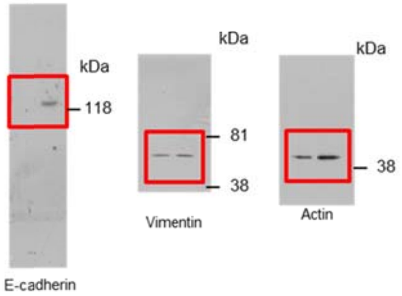

Fig. 6h

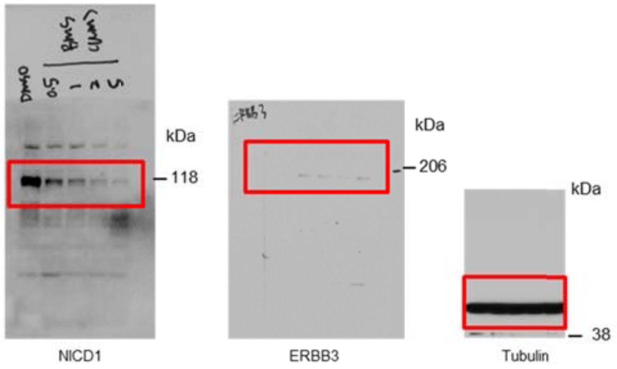

Supplementary Figure 10 (continued). Uncut Western blotting films. Red boxed are bands presented in figures.

Fig. 6g

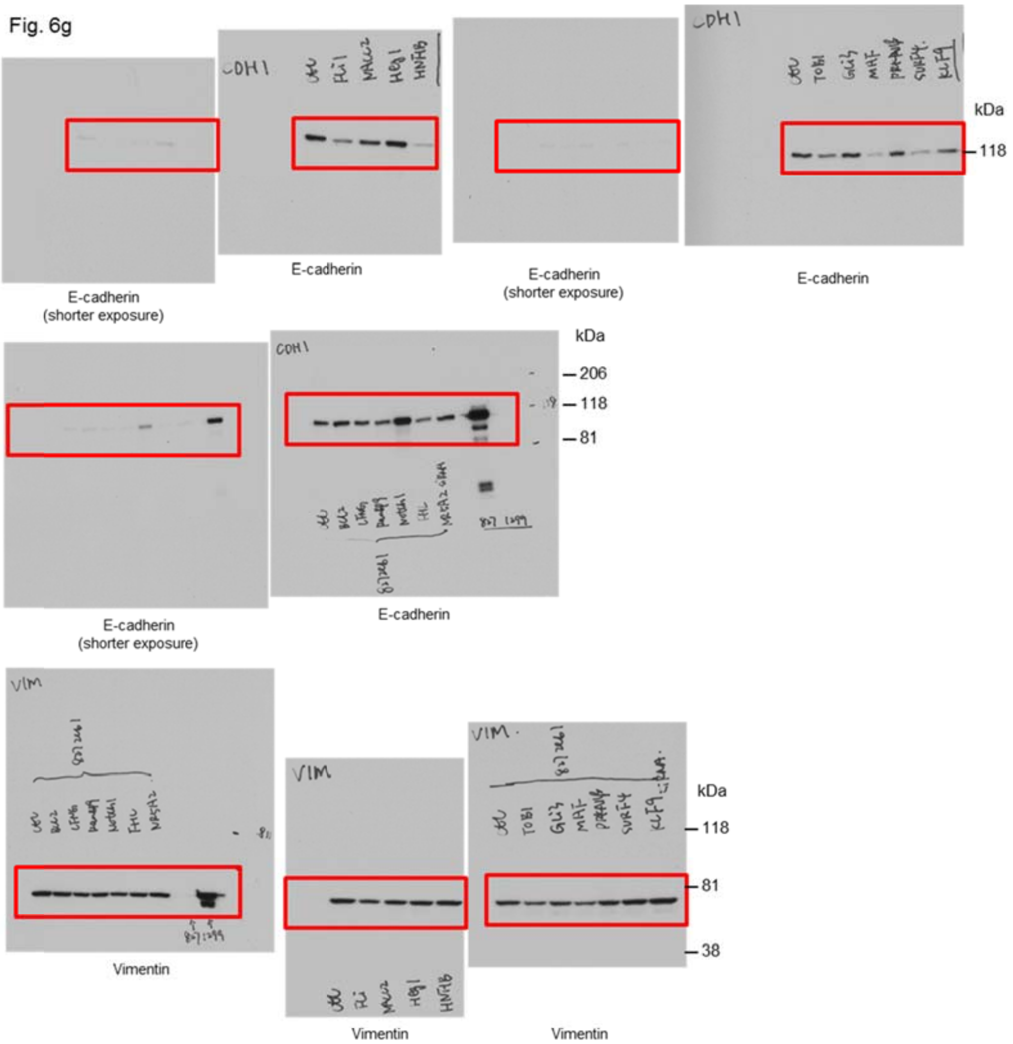

Fig. 7e

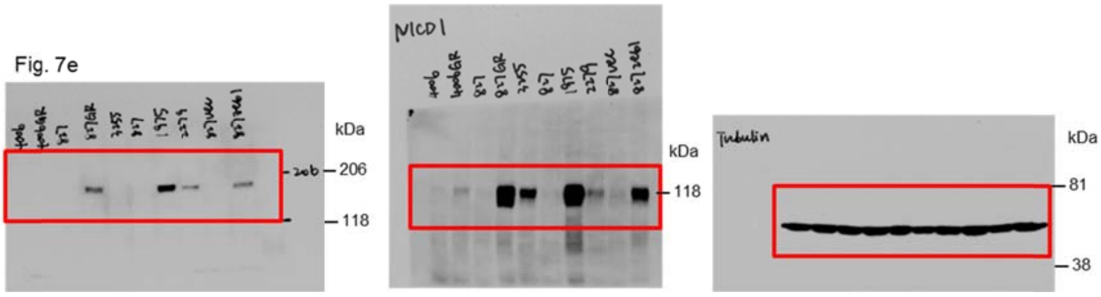

Supplementary Figure 10 (continued). Uncut Western blotting films. Red boxed are bands presented in figures.

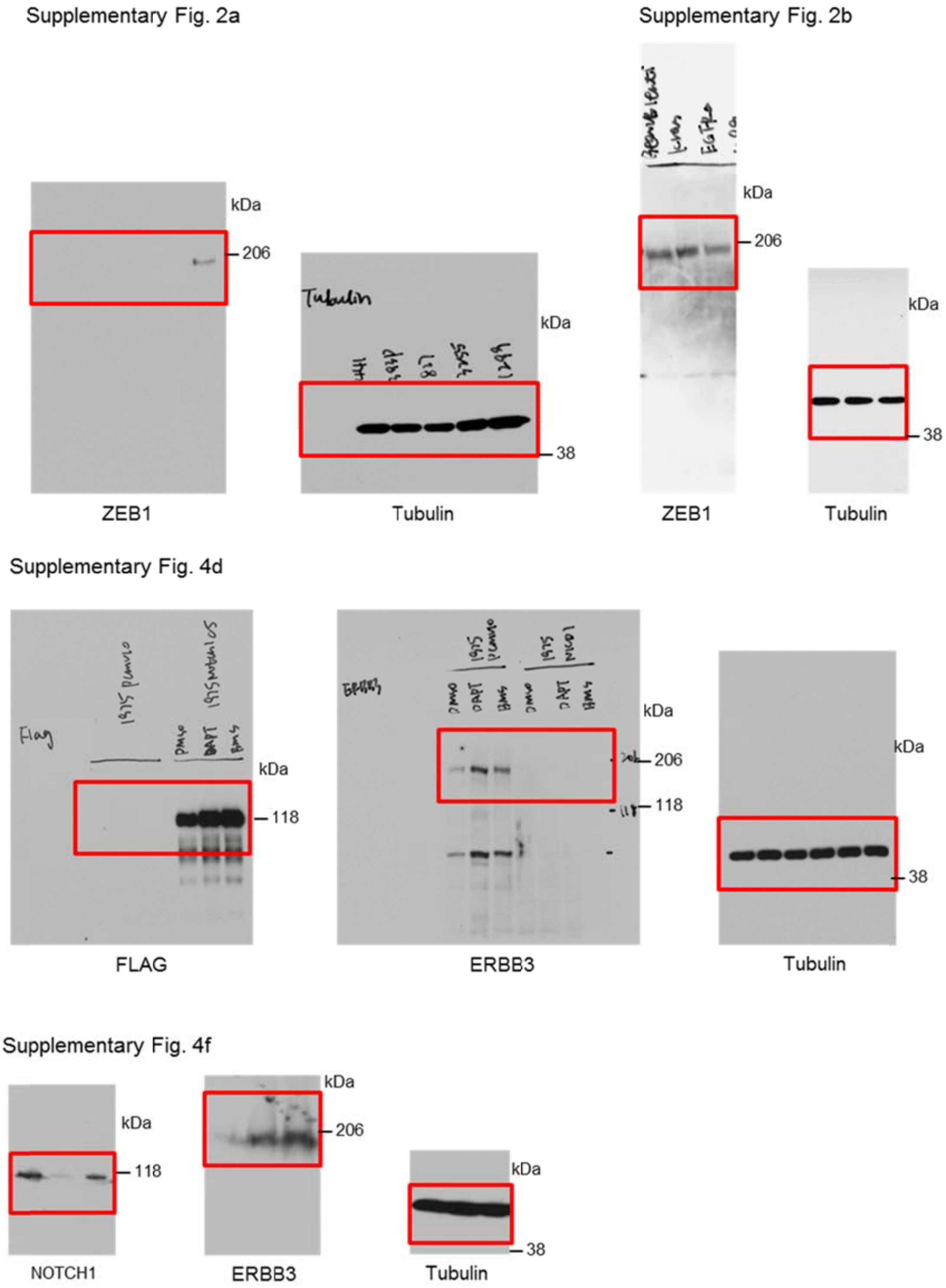

Supplementary Figure 10 (continued). Uncut Western blotting films. Red boxed are bands presented in figures.

Supplementary Table 1. Demographic and clinical information for the 41 paired frozen lung adenocarcinoma and adjacent normal lung tissues used in this study

**Total number of tissues: 41 pairs**		
**Age at surgery**	Mean	67
	Range	46-89
**Sex**	Male	22 (54%)
	Female	19 (46%)
**Race**	Caucasian	37 (90%)
	American Indian/Alaska Native	1 (2%)
	Unknown	3 (8%)
**Smoking status**	Never	16 (39%)
	Former	15 (37%)
	Current	10 (24%)
**Stage**	I	16 (39%)
	II	17 (41%)
	III	8 (20%)

Supplementary Table 2. Gene expression levels in paired frozen lung adenocarcinoma and adjacent normal lung tissues (n=41 pairs)

	**Tumour mean**	**Normal mean**	**Fold change**	**P value**
CDH1	1.67	1.03	1.62	0.007
VIM	1.04	1.77	-1.70	0.001
ZEB1	0.74	1.01	-1.36	0.035

Supplementary Table 3. Gene mutation status of lung adenocarcinomas in this study

Tumor	Mutation status	
	KRAS	*EGFR*
1 (reference 23)	wt	wt
2 (references 23, 24)	wt	L858R
3 (reference 23)	wt	wt
4 (reference 24)	wt	E746_A751del
5	wt	wt
6 (reference 23)	wt	wt
7 (references 23, 24)	Q61H	wt
8	G12R	wt
9	wt	wt
10 (references 23, 24)	wt	L858R
11 (references 23, 24)	wt	E746_A750del
12 (references 23, 24)	G12D	wt
13 (references 23, 24)	wt	E746_S752A
14 (references 23, 24)	wt	E746_A750del
15	Q61H	wt
16	G12C	wt
17	wt	E746_A751del
18	wt	wt
19	wt	wt
20	G12C	wt
21	G12D	wt
22	wt	wt
23	wt	wt
24	wt	wt
25	G12C	wt
26	wt	wt
27	G12R	wt
28	wt	wt
29	wt	wt
30	wt	wt
31	wt	wt
32	G12S	wt
33	wt	wt
34	G12R	wt
35	G12C	wt
36	wt	wt
37	wt	wt
38	wt	wt
39	G12C	wt
40	wt	wt
41	G12C	wt

Supplementary Table 4. Lung adenocarcinoma / normal lung fold changes of EGFR in the Oncomine datasets.

Dataset	*EGFR*		*KRAS*	
	**Fold change**	**p**	**Fold change**	**p**
Selamet	1.993	2.94e-10	1.278	9.86e-5
Hou	1.080	1.98e-4	1.234	7.22e-4
Landi	1.189	6.154e-4	1.266	0.001
Su	2.112	1.33e-5	1.216	0.143
Okayama	1.874	5.45e-13	1.188	3.01e-4
